# Dinuclear and mononuclear metal(II) polypyridyl complexes against drug-sensitive and drug-resistant *Plasmodium falciparum* and their mode of action

**DOI:** 10.1186/s12936-022-04406-0

**Published:** 2022-12-17

**Authors:** Jing Wei Lai, Mohd Jamil Maah, Kong Wai Tan, Rozie Sarip, Yvonne Ai Lian Lim, Rakesh Ganguly, Loke Tim Khaw, Chew Hee Ng

**Affiliations:** 1 Department of Chemistry, Faculty of Science, Universiti Malaya, 50603 Kuala Lumpur, Malaysia; 2 Department of Parasitology, Faculty of Medicine, University of Malaya, 50603 Kuala Lumpur, Malaysia; 3 Shiv Nadar University, Greater Noida, India; 4 Department of Microbiology and Immunology, School of Medicine, International Medical University, 57000 Kuala Lumpur, Malaysia; 5 Department of Pharmaceutical Chemistry, School of Pharmacy, International Medical University, 57000 Kuala Lumpur, Malaysia

**Keywords:** Copper complex, Zinc complex, Antimalarial, *Plasmodium falciparum*, Reactive oxygen species, Depolarization of mitochondrial membrane potential, Proteasome inhibition, Apoptosis

## Abstract

**Background:**

Malaria remains one of the most virulent and deadliest parasitic disease in the world, particularly in Africa and Southeast Asia. Widespread occurrence of artemisinin-resistant *Plasmodium falciparum* strains from the Greater Mekong Subregion is alarming. This hinders the national economies, as well as being a major drawback in the effective control and elimination of malaria worldwide. Clearly, an effective anti-malarial drug is urgently needed.

**Methods:**

The dinuclear and mononuclear copper(II) and zinc(II) complexes were synthesized in ethanolic solution and characterized by various physical measurements (FTIR, CHN elemental analysis, solubility, ESI-MS, UV-Visible, conductivity and magnetic moment, and NMR). X-ray crystal structure of the dicopper(II) complex was determined. The in vitro haemolytic activities of these metal complexes were evaluated spectroscopically on B+ blood while the anti-malarial potency was performed in vitro on blood stage drug-sensitive *Plasmodium falciparum* 3D7 (*Pf3D7*) and artemisinin-resistant *Plasmodium falciparum* IPC5202 (*Pf5202*) with fluorescence dye. Mode of action of metal complexes were conducted to determine the formation of reactive oxygen species using PNDA and DCFH-DA dyes, JC-1 depolarization of mitochondrial membrane potential, malarial 20S proteasome inhibition with parasite lysate, and morphological studies using Giemsa and Hoechst stains.

**Results:**

Copper(II) complexes showed anti-malarial potency against both *Pf3D7* and *Pf5202* in sub-micromolar to micromolar range. The zinc(II) complexes were effective against *Pf3D7* with excellent therapeutic index but encountered total resistance against *Pf5202*. Among the four, the dinuclear copper(II) complex was the most potent against both strains. The zinc(II) complexes caused no haemolysis of RBC while copper(II) complexes induced increased haemolysis with increasing concentration. Further mechanistic studies of both copper(II) complexes on both *Pf3D7* and *Pf5202* strains showed induction of ROS, 20S malarial proteasome inhibition, loss of mitochondrial membrane potential and morphological features indicative of apoptosis.

**Conclusion:**

The dinuclear [Cu(phen)-4,4′-bipy-Cu(phen)](NO_3_)_4_ is highly potent and can overcome the total drug-resistance of *Pf5202* towards chloroquine and artemisinin. The other three copper(II) and zinc(II) complexes were only effective towards the drug-sensitive *Pf3D7*, with the latter causing no haemolysis of RBC. Their mode of action involves multiple targets.

**Graphical Abstract:**

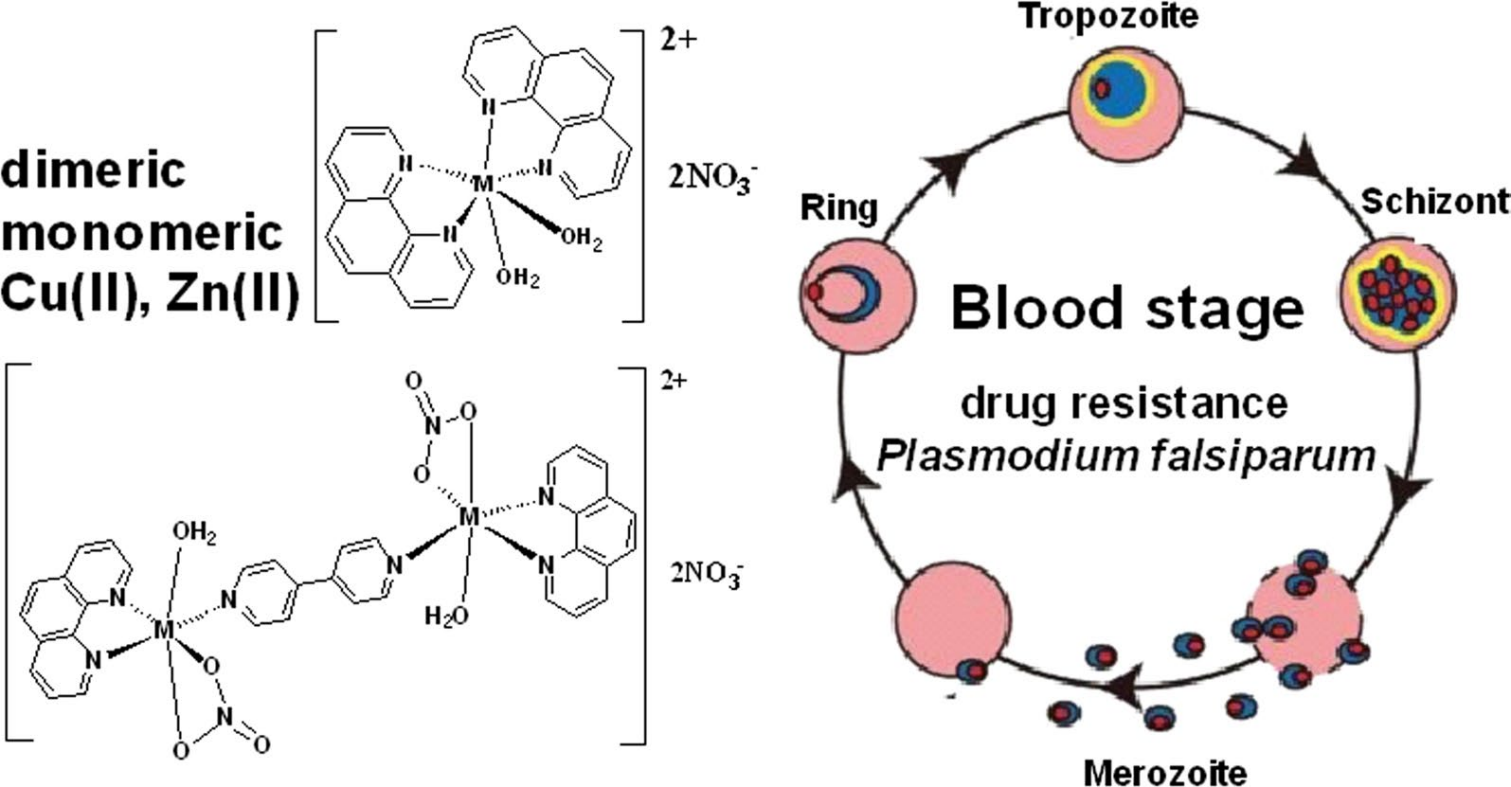

**Supplementary Information:**

The online version contains supplementary material available at 10.1186/s12936-022-04406-0.

## Background

WHO reported an estimated 241 million cases of malaria worldwide in 85 endemic countries, with estimated malaria deaths of 627,000 in 2020 [1]. The situation is worsened by the spreading of drug resistance even to artemisinin-based combination therapy (ACT), and the independent emergence of *kelch13* mutations, which is the cause of artemisinin-resistance [2]. Due to increasing knockdown of more members in the cocktail of drugs in ACT, as a result of drug resistance, there is an urgent need for discovery of new antimalarial drugs.

Development of metal complexes is an attractive alternative drug design due to the unique structural and physicochemical possibilities that cannot be attained by organic molecules, and these metallo anti-malarial drugs offers potential solution to drug resistance [3–7]. Among four mixed-ligand cationic complexes, (N-benzoyl-N′,N′-di(2-hydroxyethyl)thioureato)(4,4′-di-tert-butyl-2,2′-bipyridyl)platinum(II) chloride was strongly active against both chloroquine-sensitive and chloroquine-resistant strains of malaria [8]. Out of the list of different metal-chloroquine complexes, three of the six tested showed greater anti-malarial activity against chloroquine-resistant malaria strain [9]. In another study, the potency of one of the tested copper(II) complexes was 32 times higher than that of chloroquine, and 260 times higher than that of another antiprotozoal drug, toltrazuril, suggesting copper complexes as good candidates [3]. In comparison, a lead compound [(η^5^-C_5_R_5_)Ru(PPh_3_)(phen)][PF_6_] was only less than 2 times more potent than chloroquine in *Pf3D7*, a chloroquine/artemisinin-sensitive strain of *Plasmodium falciparum,* but it was not as good as chloroquine or dihydroartemisinin against the artemisinin-resistant strain *Pf5202* [7].

Besides copper(II) complexes, zinc complexes are attractive because of their non- or low toxicity. However, complexation of zinc(II) to bioactive ligands can enhanced their anti-malarial property, but may not be always comparable to other anti-malarial drugs [10, 11]. Thus far, anti-malarial metal complexes are mostly mononuclear. However, despite some dicopper(II) and dizinc(II) complexes known to have anticancer property, there seems no report of their anti-malarial property [12–15]. Herein, the manuscript reports the synthesis and characterization of two dinuclear metal(II) complexes (Cu and Zn) with a bioactive 1,10-phenanthroline (phen) and a non-bioactive 4,4'-bipyridine (4,4’-bipy) as bridging ligand, and their anti-malarial property, with comparison to their respective mononuclear metal(II) complexes containing two phen ligands.

## Methods

### Materials and chemicals

Commercially available starting materials or chemicals were of analytical reagent grade and of the highest purity available. All were used as received without further purification. Chemicals: 1,10-phenanthroline (Acros Organics, USA), 4,4′-bipyridine (Merck, Germany), copper(II) nitrate trihydrate (Alfa Aesar, USA) and zinc(II) nitrate hexahydrate (Acros Organics, Belgium). All glassware was thoroughly washed with diluted aqua regia, then with ultra-pure water and dried overnight in an oven. All metal(II) complexes synthesized were collected using suction filtration, washed with cold ethanol, dried in an oven, and kept in a desiccator containing silica gel before characterization. All buffer stock solutions for biological assay were autoclaved at 121 °C for 15 min.

### ***Synthesis of [M(phen)***_***2***_***](NO***_***3***_***)***_***2***_

The pair of [M(phen)_2_](NO_3_)_2_ (M = copper or zinc) were synthesized in the same manner as described herein. 1,10-phenanthroline solution was added into ethanolic solution of metal(II) nitrate salt (30 ml of 1:1 v/v water:ethanol). After being heated and stirred continuously (55 °C; 525 rpm) for 2 h, each solution was filtered and yielded precipitate on slow evaporation.

[Cu(phen)_2_](NO_3_)_2_·H_2_O (**1**) Color (% Yield): Green (70.28%). FTIR (ATR) cm^−1^: 3188 (ν_s_ (O-H) br), 3061 (*v*_*s*_ (C-H) m), 1429–1608 (*v*_*s*_ (C = C) m), phen: 850 and 720 (*v*_*s*_ (C-N-C) m). Elemental Analysis: Anal. Calc. for CuC_24_H_18_N_6_O_7_: C, 50.93%; H, 3.21%; N, 14.85%. Found: C, 50.67%; H, 3.04%; N, 15.12%. Magnetic moment, µ_eff_ in B.M.: 1.84. ESI-MS( +) in aqueous-methanol: m/z 484.8 (100%), 486.7 (49%) (calc. m/z for [^63^Cu(phen)_2_(NO_3_)]^+^, 485.3; [^65^Cu(phen)_2_(NO_3_)]^+^, 487.1).

[Zn(phen)_2_](NO_3_)_2_·2H_2_O (**2**). Color (% Yield): White (73.74%). FTIR (ATR) cm^−1^: 3180 (ν_s_ (O-H) br), 3062 (*v*_*s*_ (C-H) m), 1495–1581 (*v*_*s*_ (C = C) m), phen: 846 and 723 (*v*_*s*_ (C-N-C) m).—^1^H NMR (400 MHz, DMSO-d, δ ppm): 8.049 (4H, t, Ar-H); 8.320 (4H, s, Ar-H); 8.733 (4H, d, Ar-H); 8.945 (4H, d, Ar-H). ^13^C NMR (400 MHz, DMSO-d, δ ppm): 126.640 (C_Ar_); 128.022 (C_Ar_); 129.605 (C_Ar_); 140.284 (C_Ar_); 140.941 (-C_Ar_ = N); 149.608 (-C_Ar_-N). Elemental Analysis: Anal. Calc. for ZnC_24_H_20_N_6_O_8_: C, 49.20%; H, 3.44%; N, 14.35%. Found: C, 49.11%; H, 2.89%; N, 14.33%. Magnetic moment, µ_eff_ in B.M.: 0.30. ESI-MS( +) in aqueous-methanol: m/z 485.9 (67%), 487.9 (40%) (calc. m/z for [^64^Zn(phen)_2_(NO_3_)]^+^, 486.3;[^66^Zn(phen)_2_(NO_3_)]^+^, 488.3).

### Synthesis of dinuclear [Cu(phen)-4,4′-bipy-Cu(phen)](NO_3_)_4_·2H_2_O

[Cu(phen)](NO_3_)_2_ (0.7355 g, 2 mmol) was dissolved in 1:1 v/v water:ethanol solution and an ethanolic solution of 4,4’-bipyridine (0.1560 g, 1 mmol) was added into the copper complex solution. Ethylenediamine (66.85 µl, 1 mmol) was further added into the mixture. The mixture was heated and stirred at 50 °C, 700 rpm for 2 h. The solution was then heated in water bath for 5 h and was left for evaporation at room temperature. Dark green crystals were formed after 2 days. Crystals were filtered and recrystallized with 1:2 v/v water:ethanol solution. The recrystallized crystals were then filtered and dried in the 55 °C oven overnight.

[Cu(phen)(NO_3_)(H_2_O)-4,4′-bipy-Cu(phen)(NO_3_)(H_2_O)](NO_3_)_2_ (**3**). Color (% Yield): Green (35.69%). FTIR (ATR) cm^−1^: bipy: 1610 (*v*_*s*_ (C = C) m), 1026 (*v*_*s*_ (pyridyl in-plane bending) m), phen: 856 and 721 (*v*_*s*_ (C-N-C) m). Elemental Analysis: Anal. Calc. for Cu_2_C_34_H_28_N_10_O_14_: C, 44.02%; H, 3.04%; N, 15.10%. Found: C, 44.17%; H, 2.82%; N, 14.95%. Magnetic moment, µ_eff_ in B.M.: 1.37. ESI-MS( +) in aqueous-dimethyl sulfoxide: m/z 237.9 (100%), 382.2 (30.9%) (calc. m/z for [^63^Cu(phen)-4,4’-bipy-^63^Cu(phen)(NO_3_) + 3H]^3+^, 237.8; [^63^Cu(phen)(NO_3_)-4,4′-bipy-^63^Cu(phen)-(NO_3_)-H]^2+^, 382.2).

### Synthesis of dinuclear [Zn(phen)-4,4′-bipy-Zn(phen)](NO_3_)_4_·2H_2_O

4,4’-bypyridine (10 ml of 95% ethanol; 0.1571 g; 1 mmol) was added into ethanolic solution of zinc(II) nitrate hexahydrate (10 ml of 95% ethanol; 0.6147 g; 2 mmol). The solution was heated and stirred at 55 °C, 700 rpm for 30–45 min. 1,10-phenanthroline (10 ml 95% ethanol; 0.3604 g; 2 mmol) was dissolved and poured into the heated mixture. The solution was further heated and stirred at 55 °C, 700 rpm for 2 h. White powder started to form while stirring on a hot plate. Powder was collected by using suction filtration, washed with ice cold ethanol and dried overnight in the oven.

[Zn(phen)(NO_3_)-4,4′-bipy-Zn(phen)(NO_3_)](NO_3_)_2_·2H_2_O (**4**). Color (% Yield): White (16.64%). FTIR (ATR) cm^−1^: bipy: 1610 (*v*_*s*_ (C = C) m), 1026 (*v*_*s*_ (pyridyl in-plane bending) m), phen: 848 and 725 (*v*_*s*_ (C-N-C) m). ^1^H NMR (400 MHz, DMSO-d, δ ppm): 7.396 (4H, d, Ar-H); 8.123 (4H, t, Ar-H); 8.245 (4H, s, Ar-H); 8.698 (4H, d, Ar-H); 8.894 (4H, t, Ar-H); 9.128 (4H, d, Ar-H). ^13^C NMR (400 MHz, DMSO-d, δ ppm): 126.219–129.576 (C_Ar_); 139.893–140.436 (C_Ar_); 140.903 (= C_Ar_-N); 144.927 (-C_Ar_-N); 149.580 (-C_Ar_-C_Ar_); 151.010 (-C_Ar_ = N). Elemental Analysis: Anal. Calc. for Zn_2_C_34_H_28_N_10_O_14_: C, 43.84%; H, 3.03%; N, 15.04%. Found: C, 43.80%; H, 3.44%; N, 14.71%. Magnetic moment, µ_eff_ in B.M.: 1.06. ESI-MS( +) in aqueous-dimethyl sulfoxide: m/z 383.6 (100%) (calc. for [^64^Zn(phen)(NO_3_)-4,4′-bipy-^64^Zn(phen)(NO_3_)]^2+^, 384.2).

### Characterization of metal complexes

FTIR spectra of the metal(II) complex were recorded with a Shimadzu IRAffinity-1S over a range of 400–4000 cm^−1^. Elemental analysis of carbon, hydrogen and nitrogen was carried out on a Perkin Elmer CHNS/O 2400 Series II by Universiti Malaya (UM). NMR spectra were obtained with an FT-NMR ECX 400 (400 MHz) spectrometer using deuterated dimethyl sulfoxide (DMSO-d6) as the solvent without internal reference (Universiti Malaya).

Solubility of metal(II) complexes was determined using different type of solvents, i.e., double distilled water, 95% ethanol, methanol and dimethyl sulfoxide (DMSO). The positive-ion electrospray ionization-mass spectra (ESI-MS) of approximately 200 ng/μL of (i) mononuclear metal(II) complexes dissolved in water-methanol (1:4 v/v), and (ii) dimetal(II) complexes in water-dimethyl sulfoxide (9:1 v/v) were obtained using Thermo Finnigan LCQ mass spectrometer (National University of Singapore) by infusion method (10 μL/min) with heated capillary temperature at 60 °C and capillary voltage of 21 V.

Visible spectroscopic measurement was carried out on a Perkin Elmer Lambda 25 spectrophotometer with a 1 cm optical path quartz cuvette in the range of 200–400 cm^−1^ for ultraviolet (UV) range and 400–1000 cm^−1^ for visible (Vis) range. A EUTECH Instruments PC 2700 bench top conductivity meter was used to measure the conductivity of metal(II) complex solution. Magnetic susceptibility was determined with a Sherwood Scientific MK 1 Magnetic Susceptibility Balance at room temperature.

Metal(II) complexes (**1**) and (**2**) were dissolved in double-distilled water (dd-water) and further diluted with dd-water for characterization studies, while they were further diluted with medium for biological studies. On the other hand, metal(II) complexes (**3**) and (**4**) were dissolved in DMSO and further diluted with dd-water for characterization studies while they were diluted with medium for biological studies.

#### X-ray crystal structure determination of (3)

**Fig. 1:**
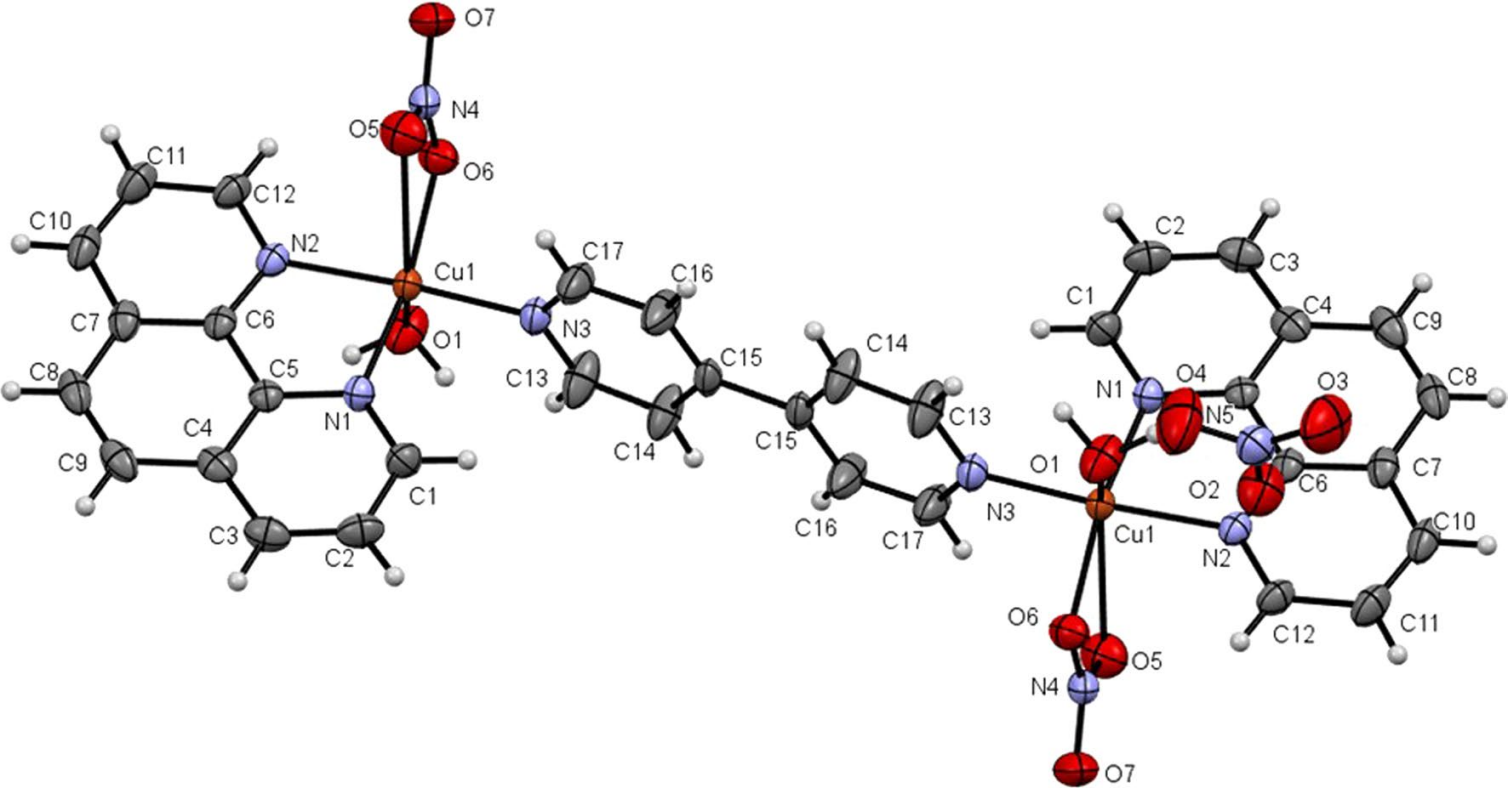
Ortep plot of dicopper(II) complex (**3**) with labelled non-hydrogen atoms (with two uncoordinated nitrate anions not shown)

Intensity data for a green block crystal, 0.40 × 0.30 × 0.20 mm of the copper(II) (**3**) was collected at 293 K (20 °C) by mounting the crystal onto quartz fibre on an Agilent Technologies SuperNova Dual diffractometer with Atlas detector, using a Supernova Cu Kα radiation (λ = 1.54184 Å). The Agilent CrysAlis PRO software was used for data acquisition, and data reduction. Structural solution was carried out with the SHELXTL suite of programs [16]. The structure was solved by direct-methods to locate the heavy atoms, followed by difference maps for the light, non-hydrogen atoms. All non-hydrogen atoms were refined with anisotropic thermal parameters and refined by a full-matrix least-squares procedure using Olex [17]. The molecular structure of complex (**3**) is shown in Fig. 1. The crystallographic data is summarized in Additional file 1: Table S1 and has been deposited at Cambridge Crystallographic Centre (CCDC No. 2158992) which can be obtained from http://www.ccdc.cam.ac.uk/Community/Request a structure/Pages/Data Request.aspx. Selected bond distances and angles are tabulated in Additional file 1: Table S2.

### Solution studies

Series of 10 mM stock solutions were prepared by dissolving the compounds in their respective solvents: phen and 4,4′-bipy in 95% ethanol; Cu(NO_3_)_2_·3H_2_O, Zn(NO_3_)_2_·6H_2_O, (**1**) and (**2**) in double distilled water; (**3**) and (**4**) in DMSO. All the stock solutions were further diluted with double distilled water to obtain 10 mL of 30 µM, 1 mM and 5 mM for their respective studies, viz. 30 µM for ultraviolet spectral studies, 1 mM for conductivity measurement and 5 mM for visible spectral studies. For the water-DMSO solutions of both complexes (**3**) and (**4**), the 30 µM, 1 mM and 5 mM solutions have 3% DMSO, 10% DMSO and 50% DMSO respectively. The absorbance at λ_max_ and conductance were of the aqueous solutions were measured at 0, 24, 48 and 72 h. The molar absorptivity and molar conductivity of the metal(II) complex solutions and their precursors were then calculated.

### Parasite culture and reagent

The drug-sensitive strain of *P. falciparum* 3D7 (*Pf3D7*) was obtained from the Department of Science, Monash University, Malaysia and cultured as previously described [18]. The artemisinin-resistant strain of *P. falciparum* IPC5202 (*Pf5202*) was obtained from BEI Resources and cultured as mentioned in the product sheet. Both strains were maintained in sealed flask at 37 °C in a humidified atmosphere of 5% O_2_, 5% CO_2_ and 90% N_2_. All buffer stock solutions for biological assays were autoclaved at 121 °C for 15 min.

### In vitro* haemolytic assay*

Fresh B+ blood was washed with RPMI1640 medium containing of 30 µg/mL gentamicin (Gibco) and centrifuged at 1000 xg for 10 min three times. The percentage of haemolysis was determined spectroscopically using 5% haematocrit containing test compound at increasing concentration and incubated for 72 h at 37 °C [19, 20]. Untreated blood acted as negative control while blood treated with sterile distilled water acted as positive control (for 100% haemolysis).

### In vitro* anti-malarial activity using SYBR Green I nucleic acid dye*

Previously reported anti-malarial assay was used with slight modification using 1% parasitaemia (ring-stage) and 5% haematocrit treated with test compounds at increasing concentration and incubated for 72 h at 37 °C [21–23]. After the incubation, the assay was terminated by freeze/thaw cycle (− 80 °C overnight before thawing for 2 h) before the addition of lysis buffer containing SYBR Green I dye into each well and mixed thoroughly using the belly dancer for 30 min in the dark. Chloroquine diphosphate salt and artemisinin were used as standard drugs. The fluorescence signal was quantified at 485 nm excitation wavelength and 528 nm emission wavelength. IC_50_ values of each test compound was determined from a dose response curve by non-linear regression analysis.

## Mechanistic studies

### ρ-nitrosodimethylaniline (PNDA) assay

A previously reported spectrometric assay using PNDA was used to quantify the amount of reactive oxygen species (ROS), viz. •OH radicals, produced by the reaction of metal(II) complexes with hydrogen peroxide in borate buffer at pH 7.5 [24–26]. The percentage of PNDA bleaching and the concentration of •OH produced were determined.

### Measurement of reactive oxygen species level using DCFH-DA

Previously published procedure with slight modification was used for the determination of intracellular ROS level in *P. falciparum* parasites [27]. Briefly, parasitized red blood cells were treated with test compounds for 24, 48 and 72 h at 37 °C. After incubation, packed cells were washed with phosphate buffer saline (PBS) solution. Subsequently, PBS solution containing 10 µM of DCFH-DA was added and incubated at 37 °C for 30 min in the dark, before the fluorescence intensities were recorded at 485 nm excitation and 535 nm emission wavelength. The data was expressed as fold change between amount of ROS produced by treated *P. falciparum* against untreated *P. falciparum*. Normalization was done with the percentage of parasite viability obtained at each condition. Hydrogen peroxide was used as positive control.

### 20S proteasome inhibition assay using parasite lysate

To harvest *P. falciparum* parasites, intraerythrocytic *Pf3D7* and *Pf5202* were collected in trophozoite and early schizont stages. Red blood cells were lysed in 0.15% (w/v) saponin lysis buffer for 10 min at 37 °C. After lysis, the parasite pellet was washed once with PBS buffer by centrifuging at 1600 rpm for 5 min. Saponin lysis buffer was added once again to remove all the red blood cells for 10 min at 37 °C, followed by washing with PBS twice and immediately used or kept at − 80 °C. To prepare parasite lysates for proteasome purification, parasite pellet was resuspended with 100 µl of pre-cooled Pierce^™^ RIPA buffer. A mild protein extraction method was used to prepare parasite extracts. The resuspended parasites were passed 20 times through a needle with a diameter of 0.4 mm in a syringe on ice to lyse the parasite. After 15 min centrifugation at 13,000 xg at 4 °C, the supernatant was removed. Protein concentration was determined by the Bradford assay [28]. The activities of 3 proteolytic sites were determined by fluorescence assay using fluorogenic peptide substrates (Suc-LLVY-AMC for chymotrypsin-like, Boc-LRR-AMC for trypsin-like and Suc-LLE-AMC for caspase-like) [24–26, 29] Proteasome activities were determined by monitoring the substrate hydrolysis for 30 min at 37 °C with 340 nm excitation and 465 nm emission wavelength. Activity of each proteolytic site were calculated, and the IC_50_ values were determined from the non-linear regression analysis. VR23 proteasome inhibitor was used as positive control.

### JC-1 mitochondrial membrane potential study

Mitochondrial membrane potential was investigated using a BD^™^ MitoScreen Flow Cytometry Mitochondrial Membrane Potential Detection JC-1 kit. In brief, parasitized red blood cells were treated with test compounds at desired concentrations for 12 and 48 h at 37 °C. After incubation, packed cells were stained as per manufacturer’s instructions [30]. Dissipation in Δψ_m_ was measured by calculating the ratio between the red and green fluorescence.

### Morphology study by Giemsa and Hoechst staining

Detections of haemolytic and apoptotic morphological features were examined on Giemsa-stained thin blood smear. The parasite was then visualised with oil immersion (100 × magnification) using upright brightfield microscope. Another thin blood smear was prepared, fixed with methanol, stained in 10 µg/mL of Hoechst 33,258 stain, and kept in the dark for 30 min at room temperature [31]. A cover slip was applied, and the nuclear morphological examination was then visualized with at 100 × magnification with oil by fluorescence microscope using DAPI filter.

## Results and discussion

### Synthesis and characterization of copper(II) and zinc(II) complexes

**Fig. 2:**
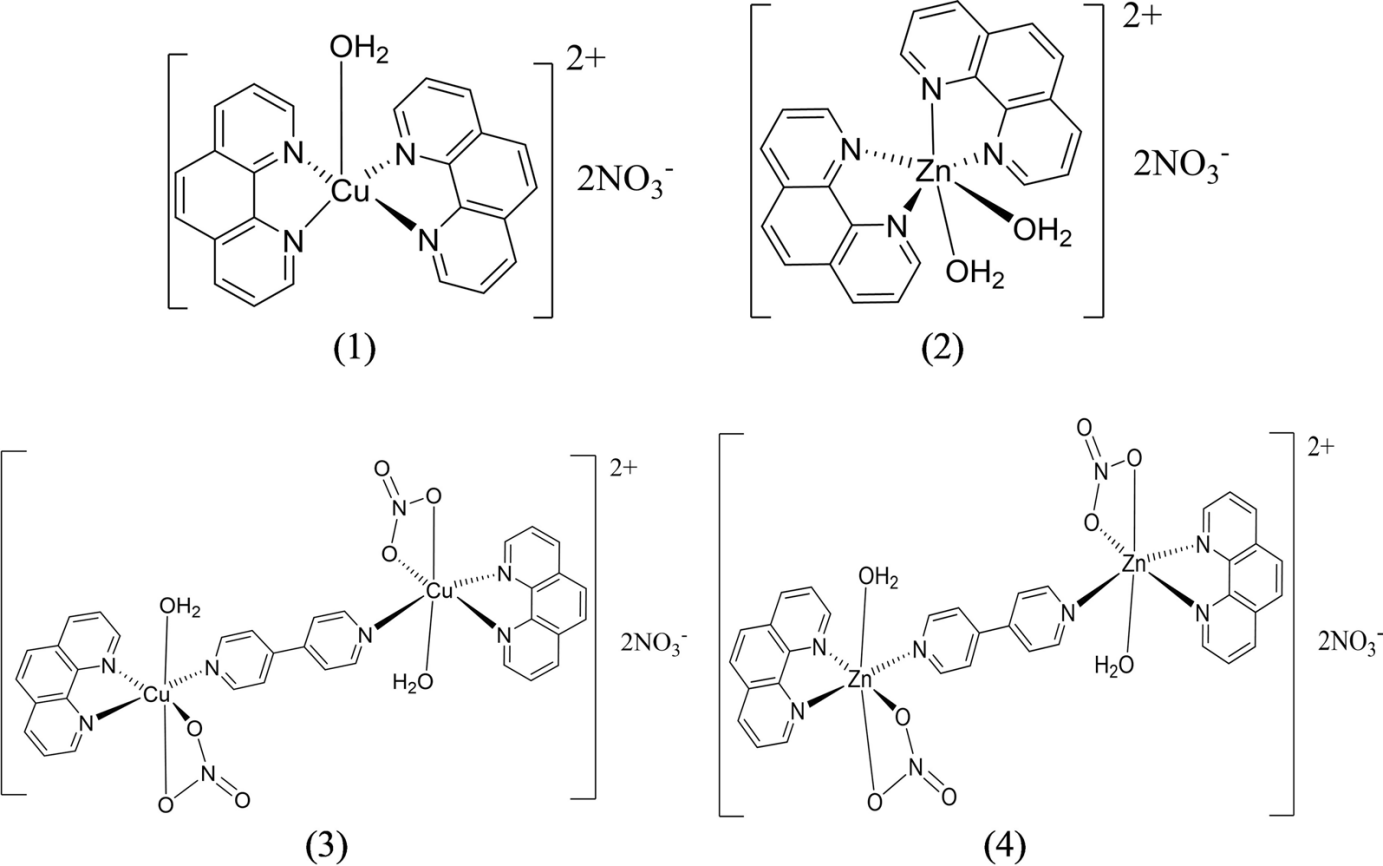
ChemDraw structures of (**1**)–(**4**) in the solid state. Structures of copper(II) complexes (**1**) and (**3**) are from their solved crystal structures whereas those of zinc(II) complexes (**2**) and (**4**) are postulated

All the [Cu(phen)_2_(H_2_O)](NO_3_)_2_ (**1**), [Zn(phen)_2_(H_2_O)_2_)](NO_3_)_2_ (**2**), [Cu(phen)(NO_3_)(H_2_O)]-4,4′-bipy-[Cu(phen)(NO_3_)(H_2_O)](NO_3_)_2_ (**3**), and [Zn(phen)(NO_3_)(H_2_O)-4,4′-bipy-Zn(phen)(NO_3_)(H_2_O)](NO_3_)_2_ (**4**) complexes contain two coordinated phen ligands, with one or two phen coordinated to the same metal atom. All solid products were remarkably air stable. The proposed formulae of (**1**)–(**4**) are supported by elemental analyses. The crystal structure of the mononuclear copper(II) complex (**1**) has been previously reported with one chelated water molecule and two phen ligands and having a distorted trigonal bipyramid geometry about the copper atom [32]. The structure of the dicopper(II) 4,4′-bipy-bridged complex (**3**) is reported herein (Fig. 1). The structure of **3** is similar to those reported previously [33, 34]. The environment about each copper(II) atom in (**3**) is a distorted octahedron, with Cu-N1(phen), Cu-N2(phen), Cu-N3(amino-4,4'-bipy), Cu-O1(OH_2_), Cu-O6(ONO_2_) and Cu-O5(ONO_2_) bond distances of 2.028, 2.016, 1.986, 2.224, 1.994 and 2.707 Ǻ (Additional file 1: Table S2). All the complexes (**1**)–(**4**) exhibit two peaks in their FTIR spectra in the range of 846–856 and 720–725 cm^−1^, which are attributed to ν(C-N-C) vibrations of the coordinated phen [35]. The presence of 4,4′-bipy in complexes (**3**) and (**4**) are indicated by two peaks at 1610 and 1026 cm^−1^ in their FTIR spectra [36]. The solid structure of the zinc(II) complex (**2**) is unknown but is most probably octahedral, like that of [Zn(phen)_2_(H_2_O)_2_]L (L = fumarate) [37]. The structure of the dizinc(II) complex (**4**) may be similar to (**3**), unlike that reported for catena-poly[diaqua(1,10-phenanthroline-κ^2^N,N′)zinc(II)]-μ-4,4′-bipyridine-κ^2^N,N’] dinitrate 4,4′-bipyridine hemisolvate monohydrate [38]. Additionally, the presence of phen and 4,4’-bipy ligands in the Zn(II) complexes (**2**) and (**4**) are validated by ^1^H- and ^13^C-NMR spectral data (Additional file 1: Figures S1.1 to S2.2). The ChemDraw structures of (**1**)–(**4**) are shown in Fig. 2.

Structurally, these set of four complexes have their central metal atoms possessing diverse structures, viz. trigonal bipyramid, octahedral and square pyramid. Additionally, the metal(II) complexes (**2**) and (**4**) also show distinct structural differences, the former is mononuclear while the latter is dinuclear, which could affect their chemical and biological properties. For example, the polymeric chain [Cu(H_2_ct)Cl]_n_ (ct, 5α-ketoglutaric acid thiosemicarbazone) complex was reported to increase DNA synthesis of cancer cells, while the dimeric [Cu(H_2_ct)Cl]_2_ decreased DNA synthesis [39]. In another report on a set of monomeric and dimeric zinc complexes of N-furfuryl-N-substituted benzyldithiocarbamate, it was found the monomeric species are more stable than the dimeric ones, and their antiproliferative effect are distinctly different [40].

### Solution structures of (1)–(4) in aqueous solution

All the complexes were very soluble in DMSO but less soluble in water, and DMSO-water solutions (low DMSO) of these complexes were suitable for various physical, chemical, and biological studies. Conductivity measurement, and UV-visible spectroscopy were used to elucidate their metal species in aqueous solutions and their stability. Absorbance at λ_max_ of the UV spectra (30 µM) and the visible spectra (5 mM), and conductivity (1 mM) of the aqueous solution of metal(II) complexes (**1**)–(**4**) and its precursors were obtained at 0, 24, 48 and 72 h.

The copper(II) and zinc(II) nitrate salts, and the metal (II) complexes (**1**) and (**2**) have molar conductivity values of about 215–271 S cm^2^ mol^−1^ (Additional file 1: Table S3), which are within the range of typical 2:1 electrolytes [41, 42]. This suggests that each molecule of the metal(II)-bis(phen) complexes (**1**) and (**2**) have dissociated into cationic [Cu(phen)_2_]^2+^ and [Zn(phen)_2_]^2+^ respectively (or exist as their hydrated species [Cu(phen)_2_(H_2_O)]^2+^ and [Zn(phen)_2_(H_2_O)_2_]^2+^) and two NO_3_^−^ ions upon dissolution in aqueous solution. The presence of these aqueous cationic species of (**1**) and (**2**) are supported by intense UV spectral peaks {222, 271 nm for (**1**); 223, 270 nm for (**2**)} (Additional file 1: Table S4) attributed to coordinated phen [43]. The visible spectral peak (λ_max_, 709 nm; ε, 64 mol^−1^dm^3^cm^−1^) of [Cu(phen)_2_(H_2_O)]^2+^ of (**1**), due to d-d transition, is characteristic of [CuL_2_]^2+^ (L = bpy, phen) species found by others [44–46]. The presence of two coordinated phen in the postulated hydrated species of (**1**) and (**2**) is supported by ESI-MS data which identifies the gaseous adduct [M(phen)_2_(NO_3_)]^+^ as the main species (Additional file 1: Table S5). Coordinated water molecules of aqueous species of (**1**) and (**2**) can easily dissociate under ESI-MS conditions and the free gaseous nitrate anion is known to easily associate or attracted to free gaseous metal cation or metal-ligand cationic species under ESI-MS conditions [47, 48]. In fact, the negative ion mode ESI-MS data shows the presence of major m/z peak corresponding to the NO_3_^−^ ions, indicating presence of these dissociated anions (data not shown). The molar conductivity and visible molar absorptivity of (**1**), and the molar conductivity of (**2**) remains practically unchanged for 24, 48 and 72 h durations, suggesting the stability of the metal(II) cationic species and no change in their coordination spheres.

Further, the dimetal(II) complexes (**3**) and (**4**) have higher molar conductivity values of 350–366 S cm^2^ mol^−1^, which are intermediate between the values for 2:1 (145–273 S cm^2^ mol^−1^) and 3:1 (408–435 S cm^2^ mol^−1^) electrolytes (Additional file 1: Table S3) [42]. This suggests two dicopper(II) species in the solution of (**3**). Similarly, the presence of coordinated 4,4’-bipy in (**3**) and (**4**) can be inferred by comparing the intense UV peak at 240 nm of free 4,4-bipy (ε, 15,333 mol^−1^dm^3^cm^−1^) with the enhanced UV peak of (**3**) at 230 nm (ε, 65,000 mol^−1^dm^3^cm^−1^) and that of (**4**) at 231 nm (ε, 78,333 mol^−1^dm^3^cm^−1^), respectively, attributed to the coordinated 4,4′-bipy (Additional file 1: Table S4). Similar to mononuclear complexes (**1**) and (**2**), the presence of coordinated phen in dinuclear metal(II) complexes (**3**) and (**4**) can be inferred from their intense UV peaks at 230 nm and 272 nm for (**3**) and those at 231 nm and 270 nm for (**4**). The ESI-MS data (Additional file 1: Table S5) of (**3**) shows two peaks at m/z values of 237.93 (100%) and 382.18 (31%) which may be assigned to the adducts [Cu(phen)-4,4′-bipy-Cu(phen)(NO_3_)]^3+^ and [Cu(phen)(NO_3_)-4,4′-bipy-Cu(phen)(NO_3_)]^2+^, respectively. The calculated value for the 31% peak is 383.23 which could be [Cu(phen)(NO_3_)-4,4'-bipy-Cu(phen)(NO_3_) + H]^2+^. As stated in the preceding paragraph, the two dinuclear metal(II) complexes, viz. dimeric [Cu(phen)(NO_3_)(H_2_O)-4,4′-bipy-Cu(phen)(NO_3_)(H_2_O)](NO_3_)_2_ (**3**), and dimeric [Zn(phen)(NO_3_)(H_2_O)-4,4′-bipy-Zn(phen)(NO_3_)(H_2_O)](NO_3_)_2_ (**4**), have coordinated nitrate ion at each metal(II). Thus, the coordinated nitrate ions in the dimetal(II) cationic species in the aqueous-DMSO (9:1 v/v) solution only dissociate partially and can thus account for their unusual molar conductivities which are intermediate between 2:1 and 3:1 electrolytes.

### Haemolysis studies of RBC

**Fig. 3:**
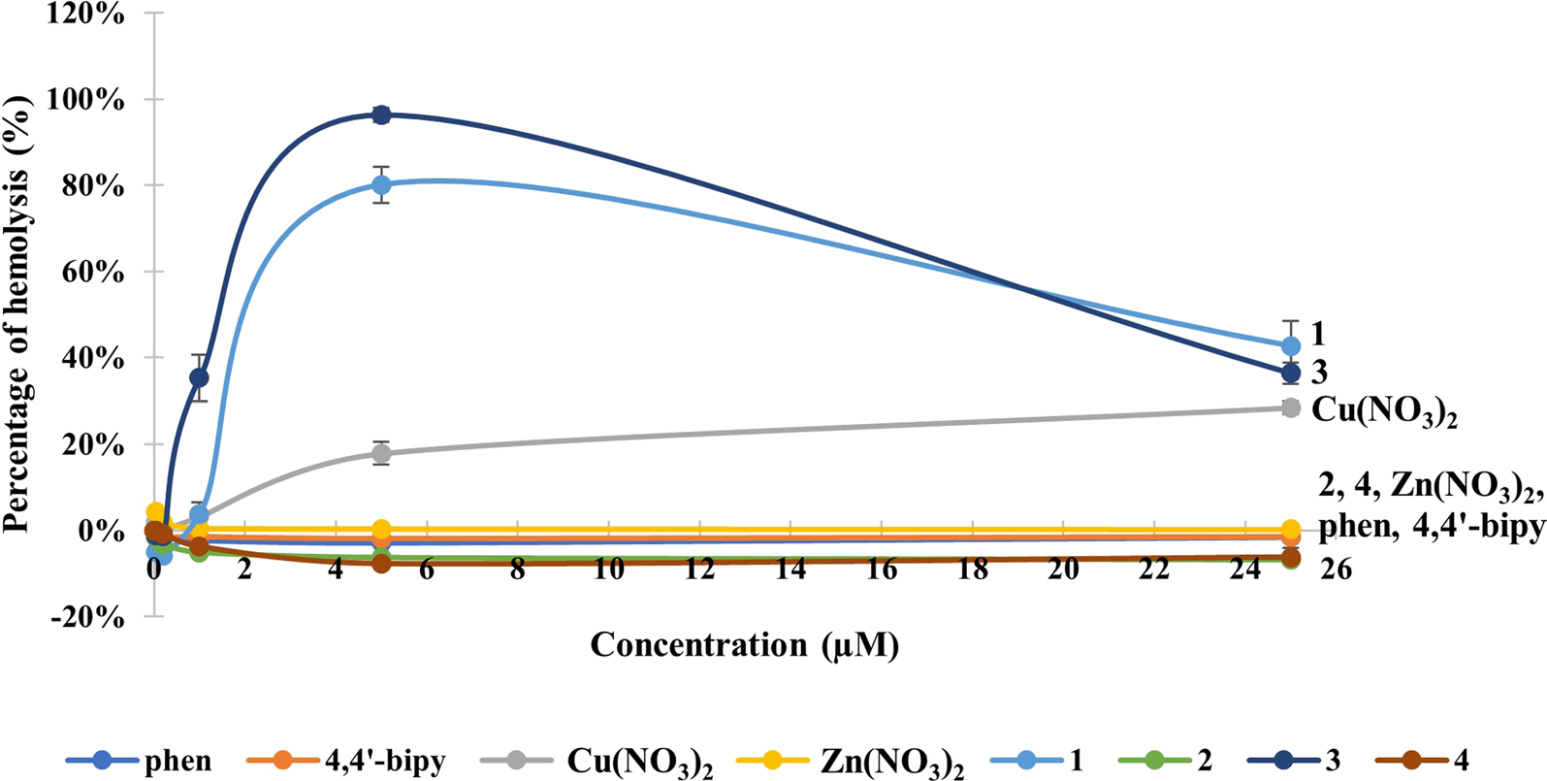
The percentage of haemolysis of (**1**)–(**4**) and their precursors after 72 h incubation

Cytotoxicity potency towards unparasitized host cells—red blood cells (RBC) are also crucial as it is commonly used to determine the safety of a pharmaceutical drug. The health of RBC is critical as they are the oxygen carriers from the lungs to bodies’ tissues which are important to the survival of the human [49]. Percentage of haemolysis, which is also known as haemolytic index (HI), was assessed spectroscopically from the volume of haemoglobin released. Dose response curve of (**1**)–(**4**) and its precursors are shown in Fig. 3.

Copper(II) complexes (**1**) and (**3**), as well as Cu(NO_3_)_2_, showed concentration dependent haemolysis with an elevation in lysis of RBC with increasing concentration. At the concentration of  ≤ 0.2 µM, copper(II) complexes did not possess any toxic effect toward RBC. At 1 µM, none to mild haemolysis were observed, with (**3**) showing the highest HI value (35%), followed by HI value of (**1**) and Cu(NO_3_)_2_ at 4–3%, respectively, indicating no haemolysis. Haemolysis of (**1**) and (**3**) reaches the max at 5 µM (80% and 96%) that is near to the 50% parasite growth inhibition. An unusual event was seen in (**1**) and (**3**) where the HI values dropped from 5 µM to 25 µM at 43–36%, respectively. This may be due to the rapid ROS-induced inhibition initiated by (**1**) and (**3**) at high concentration that led to the oxidation of iron (II) ion (Fe^2+^, orange red) in the haemoglobin into iron(IV) ion (Fe^4+^, pale violet) [50]. Thus, deteriorated the redness of haemoglobin and could not measure accurately at 570 nm. As for the ligands, and the zinc(II) complexes (**2**) and (**4**), no haemolysis was observed on B+ blood, indicating the non-toxic nature up to 25 µM. This implies that these compounds are practically safe to be used on human.

### Anti-malarial activity

The SYBR Green 1-based assay is commonly used to evaluate potential antimalarial drugs [21]. Mature RBC cells do not have DNA-containing nuclei and the SYBR Green is a cyanine dye which fluorescent very intensely when bound directly to double stranded DNA of *P. falciparum* which infects the mature RBC cells. As can be seen from the IC_50_ values (Table 1), *Pf3D7* strain was found to be sensitive to chloroquine (CQ) and artemisinin (ART) but the *Pf5202* strain was resistant to them (IC_50_ > 100 μM). The phen ligand has an IC_50_ value of 4.27 μM towards *Pf3D7*, showing it to be a bioactive ligand while the 4,4′-bipy is not. In all the metal(II) complexes, metal(II) chelation by phen causes mainly slight enhancement of their antimalarial property except (**3**) where there is about 5 × enhancement. The copper(II) nitrate was moderately active towards both the *Pf3D7* (IC_50_, ~ 46 μM) and the drug resistant *Pf5202* (IC_50_, ~ 49 μM) malaria strains while the zinc(II) nitrate was not active towards both strains. As expected, the copper(II) complexes (**1**) and (**3**) are more cytotoxic towards the drug-sensitive strain *Pf3D7* than the corresponding zinc(II) analogues (**2**) and (**4**). This could be easily understood from the established characteristic of copper(II) complexes having the ability to induce ROS stress, inhibit growth and initiate cell death [51, 52]. However, as can be seen later, the above zinc(II) complexes do not cause any haemolysis of the RBC at high concentration but are about equally effective against the drug-sensitive malaria strain *Pf*3D7.

**Table 1:** IC_50_ of (**1**)–(**4**) and its precursors for *Plasmodium falciparum 3D7* (*Pf3D7*) and *IPC5202* (*Pf5202*) at 72 h incubation

Compound	IC_50_ of *Pf3D7* (µM)	IC_50_ of *Pf5202* (µM)
phen	4.27 ± 0.05	> 100
4,4-bipy	> 100	> 100
Cu(NO_3_)_2_·3H_2_O	46.10 ± 4.29	49.37 ± 2.30
Zn(NO_3_)_2_·6H_2_O	> 100	> 100
**1**	3.51 ± 0.47	17.87 ± 2.73
**2**	4.44 ± 0.53	> 100
**3**	0.90 ± 0.12	2.21 ± 0.02
**4**	3.93 ± 0.18	> 100
CQ	0.03 ± 0.01	> 100
ART	0.04 ± 0.01	> 100

The mononuclear copper(II) complex [Cu(phen)_2_(H_2_O)](NO_3_)_2_ (**1**) was slightly more potent than phen towards *Pf3D7* but its potency towards the drug-resistant *Pf5202* malaria strain was reduced by threefold, i.e. encountering only slightly drug-resistant. The dicopper(II) complex (**3**), with IC_50_ value of 0.9 μM, was much more potent than phen (IC_50_ 4.27 μM) towards *Pf3D7* (by 4.7x) and was still effective against the *Pf5202* (IC_50_, 2.2 μM), suggesting its potential use against drug-resistant malaria. On the other hand, the dizinc(II) complex (**4**) was potent towards the drug-sensitive *Pf3D7* (IC_50_, 3.9 μM) but it encountered total drug resistance with the artemisinin-resistant *Pf5202* malaria strain (IC_50_ > 100 μM). From the above analysis, it seems that (**3**) is highly promising and has good potential against drug-resistant malaria when we see that both clinical drugs, chloroquine and artemisinin, encountered a massive drop in efficacy of more than 3000 × and the *Pf5202* was totally resistant towards these two drugs. In comparison, another highly promising chemotype, a “half-sandwich” cyclopentadienylruthenium(II) lead compound Ru2, exhibited fast parasiticidal activity against both ring and trophozoite stages of a synchronized *Pf3D7* strain and was highly potent against *Pf5202* resistant strain (IC_50_, 0.068 μM) [7]. Surprisingly, the most potent Fe(III)-multidentate ligand complex was more potent towards different resistant malaria strains (IC_50_, 30–50 μM) than towards chloroquine-sensitive strain *Pf3D7* (IC_50_, 90 μM) [53]. In our case, the copper(II) complex (**3**) was the reverse, i.e. more potent towards the sensitive strain *Pf3D7* than *Pf5202*.

### Modes of action of metal complexes

One mechanism of action of anti-malarial agents is induction of ROS and consequent oxidative stress to kill the malaria parasite. To investigate this, a relevant model reaction using PNDA as monitoring agent [24–26] and an intracellular DCFH-DA assay was used [27].

### Reactive oxygen species

#### PNDA assay

**Fig. 4:**
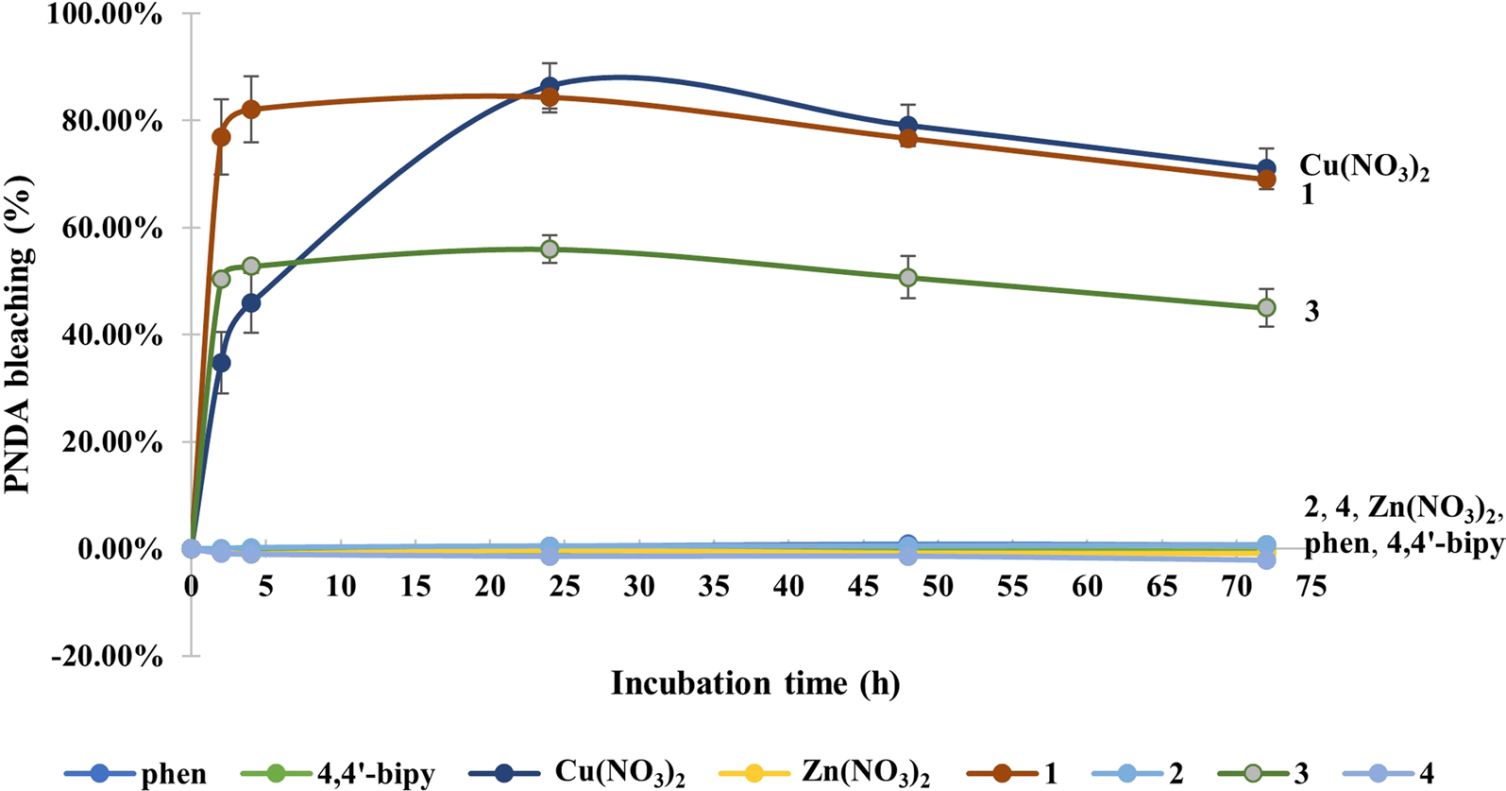
Plot of percentage PNDA bleaching of reaction mixtures, consisting of 42 µM PNDA, 60 mM of H_2_O_2_ and 30 µM of (**1**)–(**4**) and its precursors in borate buffer at pH 7.5, versus time in hour

It is now well established that numerous antimalarial compounds cause malaria death by inducing ROS and oxidative stress [54, 55]. Thus, it is of importance to be able to quantify and compare the amount of generated ROS, especially the more harmful hydroxyl radicals. For redox-active metal complexes, the PNDA assay is most appropriate as hydrogen peroxide in human cells and malaria parasites is ubiquitous [56, 57]. The amount of **·**OH radicals, produced from the reaction of the equimolar amount of test compounds with excess H_2_O_2_, is directly proportional to the bleaching of the PNDA (Fig. 4) in a 1:1 mol ratio, and the concentration of **·**OH radicals produced are tabulated in Table 2. Zinc(II) complexes (**2**) and (**4**) as well as starting materials, viz. phen, 4,4′-bipy and zinc nitrate did not generate any **·**OH radicals. All the copper(II) compounds, ie. Cu(NO_3_)_2_, (**1**) and (**3**), generated **·**OH radicals which increased from 2 to 4 h and reached a maximum at about 24 h. Overall, mononuclear (**1**) generated higher amount of **·**OH radicals than dinuclear (**3**). It was believed that (**3**) produced lesser **·**OH radicals than Cu(NO_3_)_2_ and (**1**) due to the steric hinderance because of its bulky structure with the formula of [Cu(phen)-4,4′-bipy-Cu(phen)]^4+^ in the solution. Dinuclear (**3**) might have difficulty of forming Cu(II)-hydroperoxide intermediate as each Cu(II) ion was considered as coordinately saturated, especially if the labile water molecules and nitrate ions remained coordinated. Although the mononuclear copper(II) complex (**1**) is better than the dinuclear copper(II) complex (**3**) in generating **·**OH radicals, the former is less toxic to the malaria parasite than the latter with its IC_50_ value been about 4 × larger. This suggests involvement of other mechanisms of action, as pointed out by others [51].

**Table 2:** The concentration of hydroxyl (**•**OH) radical produced by reacting 30 µM of (**1**)–(**4**) and its precursors with excess H_2_O_2_

Compound	Concentration of •OH radical (µM)				
	2 h	4 h	24 h	48 h	72 h
phen	− 0.33 ± 0.25	− 0.34 ± 0.32	0.15 ± 0.07	0.36 ± 0.17	0.30 ± 0.23
4,4′-bipy	− 0.30 ± 0.04	− 0.27 ± 0.09	− 0.18 ± 0.11	− 0.11 ± 0.26	− 0.22 ± 0.40
Cu(NO_3_)_2_·3H_2_O	14.59 ± 2.39	19.27 ± 2.36	36.29 ± 1.80	33.20 ± 1.62	29.82 ± 1.60
Zn(NO_3_)_2_·6H_2_O	− 0.24 ± 0.21	− 0.41 ± 0.22	− 0.13 ± 0.31	− 0.34 ± 0.34	− 0.37 ± 0.51
**1**	32.20 ± 2.93	34.36 ± 2.62	35.43 ± 1.22	32.19 ± 0.52	28.96 ± 0.38
**2**	0.01 ± 0.08	0.08 ± 0.03	0.20 ± 0.16	0.21 ± 0.05	0.28 ± 0.08
**3**	21.13 ± 0.41	22.16 ± 0.36	23.51 ± 1.09	21.29 ± 1.67	18.89 ± 1.45
**4**	− 0.38 ± 0.16	− 0.44 ± 0.14	− 0.60 ± 0.23	− 0.60 ± 0.53	− 0.92 ± 0.49

#### DCFH-DA assay

**Fig. 5:**
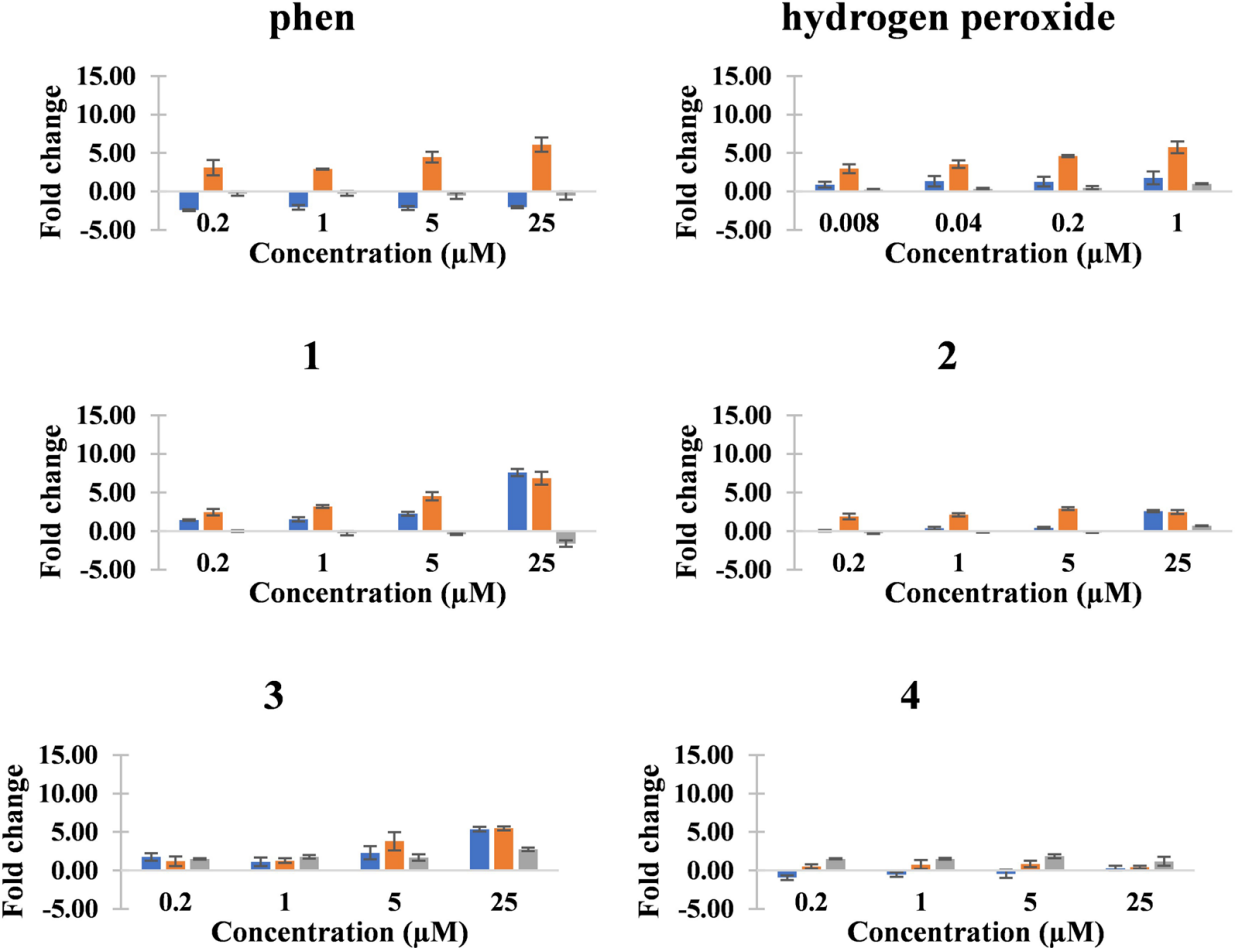
The fold changes in reactive oxygen species (ROS) induced by phen, H_2_O_2_ and (**1**)–(**4**) on *Pf3D7*. Blue (24 h). Orange (48 h). Grey (72 h)

The malaria-infected RBC cells were incubated with increasing concentration of each metal(II) complexes, (**1**)–(**4**), phen or H_2_O_2_ for 24, 48 and 72 h. The results for RBC cells infected with *Pf3D7* (Table 3 and Fig. 5) show that 48 h incubation was the most suitable as the number of fold increase in ROS was highest. Like the PNDA assay results, mononuclear copper(II) complex [Cu(phen)_2_(H_2_O)](NO_3_)_2_ (**1**) was found to be a better generator of intracellular ROS than the dinuclear [Cu(phen)(NO_3_)(H_2_O)]-4,4’-bipy-[Cu(phen)(NO_3_)(H_2_O)](NO_3_)_2_ (**3**). However, (**1**) is less potent than (**3**) against *Pf3D7* malaria strain (Table 1 IC_50_ values). Therefore, the higher anti-malarial potency of (**3**) cannot originate solely from this ROS induced inside RBC cells, and other modes of action is implicated as alluded to in the previous section [51].

**Table 3:** The fold change in ROS induced by phen and (**1**)–(**4**) on *Pf3D7*. Hydrogen peroxide (H_2_O_2_) is used as positive control

Compound	24 h				48 h				72 h			
	0.2 µM	1.0 µM	5.0 µM	25.0 µM	0.2 µM	1.0 µM	5.0 µM	25.0 µM	0.2 µM	1.0 µM	5.0 µM	25.0 µM
phen	− 2.41 ± 0.12	− 2.04 ± 0.29	− 2.15 ± 0.25	− 2.02 ± 0.15	3.10 ± 0.98	2.91 ± 0.05	4.47 ± 0.71	6.09 ± 0.91	− 0.26 ± 0.28	− 0.25 ± 0.31	− 0.55 ± 0.42	− 0.53 ± 0.52
**1**	1.44 ± 0.10	1.54 ± 0.26	2.28 ± 0.25	7.59 ± 0.46	2.45 ± 0.41	3.20 ± 0.19	4.54 ± 0.52	6.83 ± 0.83	0.00 ± 0.10	− 0.27 ± 0.27	− 0.41 ± 0.11	− 1.61 ± 0.44
**2**	0.07 ± 0.08	0.39 ± 0.19	0.45 ± 0.10	2.61 ± 0.12	1.9 ± 0.38	2.13 ± 0.21	2.95 ± 0.18	2.48 ± 0.26	− 0.27 ± 0.08	− 0.12 ± 0.05	− 0.19 ± 0.04	0.70 ± 0.05
**3**	1.76 ± 0.47	1.13 ± 0.56	2.30 ± 0.86	5.36 ± 0.30	1.20 ± 0.64	1.28 ± 0.31	3.80 ± 1.18	5.46 ± 0.25	1.49 ± 0.10	1.77 ± 0.24	1.68 ± 0.41	2.74 ± 0.24
**4**	− 0.94 ± 0.29	− 0.53 ± 0.30	− 0.44 ± 0.53	0.31 ± 0.31	0.52 ± 0.26	0.77 ± 0.58	0.85 ± 0.40	0.42 ± 0.19	1.52 ± 0.07	1.52 ± 0.14	1.85 ± 0.27	1.19 ± 0.57

Positive control	24 h				48 h				72 h			
	8 µM	40 µM	200 µM	1000 µM	8 µM	40 µM	200 µM	1000 µM	8 µM	40 µM	200 µM	1000 µM
H_2_O_2_	0.90 ± 0.37	1.34 ± 0.67	1.28 ± 0.62	1.77 ± 0.85	2.97 ± 0.57	3.55 ± 0.9	4.61 ± 0.15	5.75 ± 0.76	0.25 ± 0.08	0.34 ± 0.12	0.49 ± 0.22	1.00 ± 0.06

**Fig. 6:**
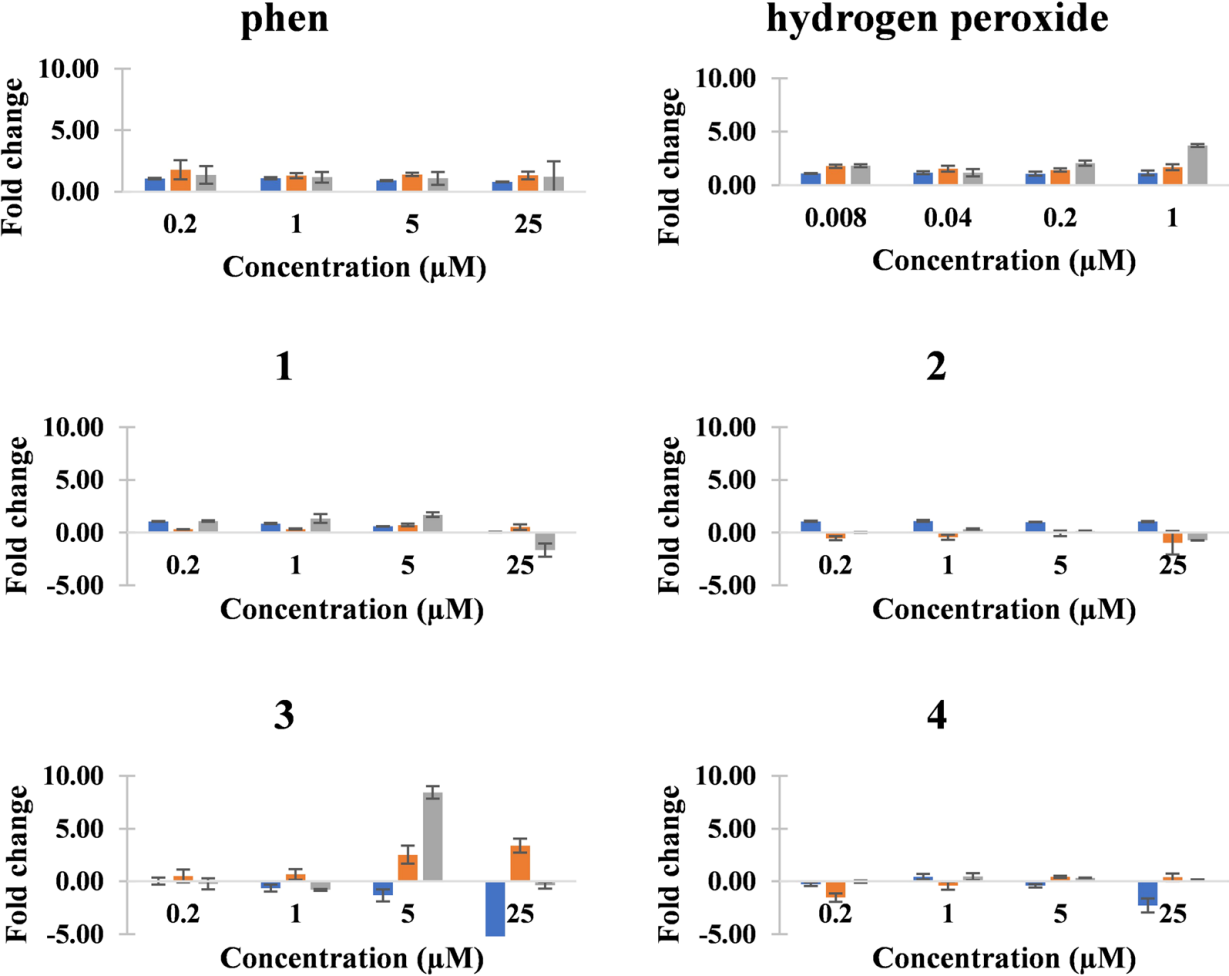
The fold changes in reactive oxygen species (ROS) induced by phen, H_2_O_2_ and (**1**)–(**4**) on *Pf5202*. Blue (24 h). Orange (48 h). Grey (72 h)

As for the *Pf5202*-infected RBC cells treated with increasing concentration of metal complexes, the best incubation duration for the treatment of *Pf5202*-infected RBC cells was still 48 h (Table 4 and Fig. 6). Among the four complexes, the two zinc(II) complexes, viz. (**2**) and (**4**), seemed not to generate any ROS as the fold increases in ROS were negative. On the other hand, the two copper(II) complexes, viz. (**1**) and (**3**), induced increasing fold change in ROS with increasing concentration of the complexes. Unlike the results for *Pf3D7*, the dicopper(II) complex (**3**) was a better generator of intracellular ROS than (**1**) in the drug-resistant *Pf5202* malaria strain and this could be correlated with its high potency towards *Pf5202* strain. This suggests dicopper(II) complexes have more potential to overcome drug-resistant malaria strains. Further detailed investigation into the mechanism of the ROS-induced mode of action of (**3**) has been planned, and it may involve targeting and damaging the redox-related proteins, and thereby disrupting redox homeostasis and processes as was found for alkyl radical-generating peroxide anti-malarial drugs, such as artemisinin [58]. Due to a recent finding, (**1**) and (**3**), as ROS inducer, will also be tested to see whether they could increase the potency of peroxide-containing anti-malarials, such as artemisinin, by direct oxidation of oxyhaemoglobin to methaemoglobin and then to iron(III)-containing hemichromes which then activate the peroxide moiety to generate carbon-centred radicals to induce death of the erythrocytic malaria [59]. These two copper(II) complexes may also be similar to metalloporphyrins which were recently found to enhance the antimalarial property of artemisinin and synthetic endoperoxide anti-malarial drugs [60]. In addition to oxidative stress as a mode of action in this section, proteasome inhibition and targeting the malarial mitochondria are presented in the next two sections.

**Table 4:** The fold change in ROS induced by phen and (**1**)–(**4**) on *Pf5202*. Hydrogen peroxide (H_2_O_2_) is used as positive control

Compound	24 h				48 h				72 h			
	0.2 µM	1.0 µM	5.0 µM	25.0 µM	0.2 µM	1.0 µM	5.0 µM	25.0 µM	0.2 µM	1.0 µM	5.0 µM	25.0 µM
phen	1.06 ± 0.06	1.08 ± 0.09	0.91 ± 0.05	0.80 ± 0.04	1.78 ± 0.78	1.31 ± 0.21	1.40 ± 0.13	1.32 ± 0.31	1.35 ± 0.71	1.17 ± 0.44	1.08 ± 0.51	1.21 ± 1.25
**1**	1.04 ± 0.03	0.86 ± 0.04	0.59 ± 0.02	0.04 ± 0.03	0.32 ± 0.02	0.33 ± 0.05	0.72 ± 0.12	0.52 ± 0.26	1.09 ± 0.07	1.33 ± 0.41	1.69 ± 0.22	− 1.66 ± 0.63
**2**	1.07 ± 0.05	1.10 ± 0.09	1.00 ± 0.02	1.04 ± 0.06	− 0.54 ± 0.19	− 0.44 ± 0.25	− 0.07 ± 0.27	− 0.97 ± 1.12	0.00 ± 0.03	0.34 ± 0.04	0.16 ± 0.04	− 0.72 ± 0.02
**3**	0.04 ± 0.33	− 0.64 ± 0.33	− 1.32 ± 0.57	− 7.33 ± 0.59	0.52 ± 0.60	0.66 ± 0.50	2.53 ± 0.85	3.39 ± 0.66	− 0.24 ± 0.52	− 0.79 ± 0.09	8.43 ± 0.60	− 0.38 ± 0.32
**4**	− 0.27 ± 0.19	0.44 ± 0.25	− 0.41 ± 0.15	− 2.29 ± 0.66	− 1.53 ± 0.39	− 0.42 ± 0.38	0.43 ± 0.09	0.39 ± 0.36	− 0.03 ± 0.11	0.45 ± 0.32	0.33 ± 0.01	0.13 ± 0.05

Positive control	24 h				48 h				72 h			
	8 µM	40 µM	200 µM	1000 µM	8 µM	40 µM	200 µM	1000 µM	8 µM	40 µM	200 µM	1000 µM
H_2_O_2_	1.10 ± 0.04	1.16 ± 0.13	1.08 ± 0.18	1.15 ± 0.22	1.78 ± 0.16	1.54 ± 0.28	1.41 ± 0.16	1.68 ± 0.28	1.82 ± 0.15	1.17 ± 0.36	2.06 ± 0.25	3.71 ± 0.14

#### Proteasome inhibition

**Fig. 7:**
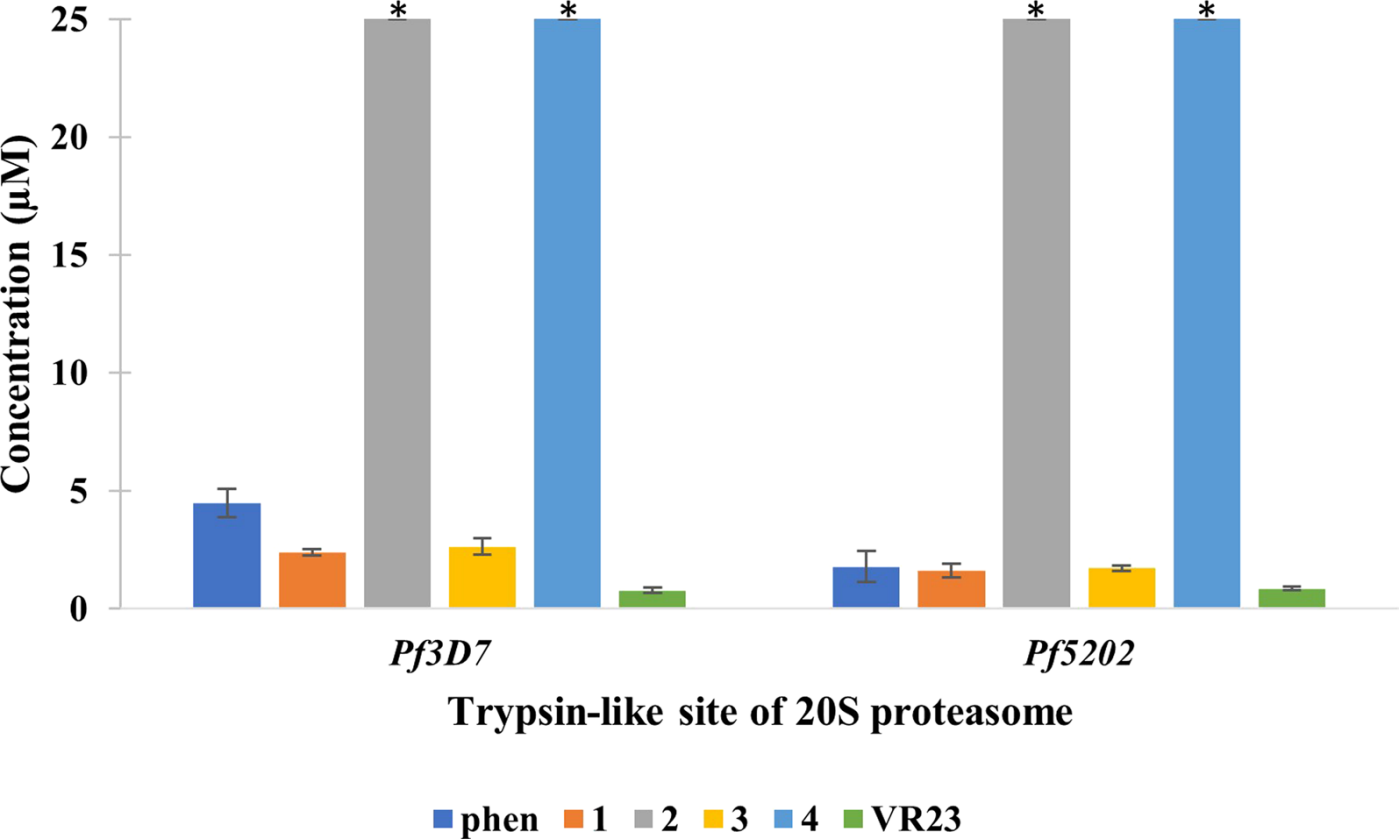
IC_50_ of phen and (**1**)–(**4**) on 20S proteasome at trypsin-like (T-L) site after incubated with respective substrate in CO_2_ incubator for 30 min. *means  > 25 µM

There is increasing concern towards occurrence and spread of drug-resistance malaria strains, and many approaches are developing to overcome drug-resistance. In fact, more results are surfacing to show the potential of developing potent parasite-specific drugs by targeting malaria proteasome based on the distinct differences between malaria parasite and the human host [61]. Recently, it was found that a tripeptide vinyl sulfone, which selectively inhibited the T-L site of *P. falciparum* 20S proteasome, could attenuate parasite growth in vivo without appreciably harming the host [62]. The basis of not harming the host was attributed to poor inhibition of the CT-L site of the human 20S proteasome. In another report, a proteasome inhibitor lactacystin inhibited development of the exoerythrocytic and erythrocytic stages of the malaria parasite [63]. As such, this section examines the inhibition of the three proteolytic sites of the 20S proteasome of the *Pf3D7* and *Pf5202* lysates with comparison to a known T-L site selective inhibitor (VR23), and the results are shown in Table 5 and Fig. 7.

**Table 5:** IC_50_ of phen and (**1**)–(**4**) that have antimalarial property on 20S proteasome at chymotrypsin-like (CT-L) site, trypsin-like (T-L) site and caspase-like (C-L) site after incubated with respective substrate in CO_2_ incubator for 30 min

Compound	IC_50_ of *Pf3D7* (µM)			IC_50_ of *Pf5202* (µM)		
	CT-L	T-L	C-L	CT-L	T-L	C-L
phen	> 25	4.47 ± 0.60	> 25	> 25	1.79 ± 0.66	> 25
**1**	> 25	2.39 ± 0.13	> 25	> 25	1.62 ± 0.30	> 25
**2**	> 25	> 25	> 25	> 25	> 25	> 25
**3**	> 25	2.64 ± 0.35	> 25	> 25	1.72 ± 0.13	> 25
**4**	> 25	> 25	> 25	> 25	> 25	> 25
VR23	> 5	0.78 ± 0.11	> 5	> 5	0.86 ± 0.09	> 5

As expected, VR23 selectively inhibited the T-L proteolytic site in both *Pf3D7* and *Pf5202* malaria strains. Interestingly, the IC_50_ values of VR23 for both malaria strains are practically the same. The free phen ligand also selectively inhibited the T-L of both strains of the parasite. Interestingly, phen is nearly 2.5 × better at inhibiting the T-L site of the drug-resistance *Pf5202* strain than that of *Pf3D7*. The two zinc(II) complexes, (**2**) and (**4**), poorly inhibited all three proteolytic sites of the proteasome. However, the two copper(II) complexes, (**1**) and (**3**), inhibited the T-L site of the *Pf3D7* more selectively than the other two sites (i.e. C-L and C-L) with both having similar IC_50_ values of about 2.5 μM. Although these complexes behave in the same way towards drug-resistant *Pf5202* strain, their IC_50_ values became about 1.5 × lower, showing that they are better at inhibiting the T-L site of this strain than that of the drug-sensitive *Pf3D7* strain. This is advantageous as malaria parasite can be harmed by such selective T-L site inhibition and can potentially overcome drug resistance in malaria. By comparing the in vitro anti-malarial assay results for *Pf5202*, (**3**) (IC_50_, 2.2 μM) is more potent than that of (**1**) (IC_50_, 17.8 μM). This suggests that the anti-malarial property of the copper(II) complexes towards *Pf5202* do not depend largely on the selective inhibition of the T-L site. Another perplexing observation is that complexes (**1**), (**3**) and phen show significant increase in efficiency in inhibiting the T-L site of *Pf5202* compared to that of *Pf3D7* (by a factor of 1.5x–2.5x) but they encounter different degree of drug-resistance in *Pf5202* (by having higher anti-malarial assay IC_50_ values for *Pf5202* than those for *Pf3D7* (by a factor of 3-20x). The extra sensitivity of drug resistant *Pf5202* strain towards (**1**), (**3**) and phen, compared to drug-sensitive *Pf3D7*, is the first instance for known anti-malarial metallodrugs. The extra sensitivity of the drug resistant *Pf5202* towards T-L selective inhibitor is unknown, but its origin may be similar to a mutant malaria strain that was resistant to a C-T selective inhibitor and had a point mutation in the noncatalytic β6 proteasome subunit [64]. This recent study on the malaria-specific C-T inhibitor, asparagine ethylenediamine, shows that C-T inhibition is not optimal and needs co-inhibition of the T-L activity (i.e. β5 site) [64].

#### Mitochondrial membrane potential

One of the mechanisms of action of some anti-malarial drugs, such as the artemisinins, is depolarisation of the mitochondrial membrane which then trigger apoptosis of the parasite [65–67]. JC-1 membrane potential assay was used to evaluate the mitochondrial membrane potential (Δψ_m_) of *Pf3D7* and *Pf5202* treated with different concentrations (1, 5, 25 μM) of copper(II) complexes (**1**) and (**3**) for 12 h and 48 h [68] and the results are shown in Table 6. Untreated healthy malaria parasite has high Δψ_m_. Generally, both copper(II) complexes caused a decrease in mitochondrial membrane potential in both malarial strains with increasing concentration of complexes for both incubation periods. At 1 μM, both (**1**) and (**3**) induced significant drop in Δψ_m_ for both *Pf3D7* and *Pf5202* strains, suggesting the collapse of mitochondrial membrane. Therefore, depolarisation of the malaria mitochondrial membrane potential is a contributing mechanism of action of both copper(II) complexes and is commonly accepted as part of the event leading to apoptotic-like death of the malaria.

**Table 6:** Mitochondrial membrane potential (Δψ_m_) of *Pf3D7* and *Pf5202* treated with different concentration of (**1**) and (**3**) for 12 h and 48 h

	Mitochondrial membrane potential (Δψ_m_)							
	*Pf3D7*				*Pf5202*			
	Complex **1**		Complex **3**		Complex **1**		Complex **3**	
	12 h	48 h	12 h	48 h	12 h	48 h	12 h	48 h
Untreated	35.1 ± 7.3	62.8 ± 23.2	35.1 ± 7.3	62.8 ± 23.2	20.0 ± 0.8	19.5 ± 3.1	20.0 ± 0.8	19.5 ± 3.1
1 µM	17.2 ± 12.8	41.1 ± 5.1	24.2 ± 1.6	45.0 ± 26.4	18.9 ± 2.8	10.4 ± 3.1	17.5 ± 2.2	19.2 ± 8.4
5 µM	29.7 ± 12.1	43.6 ± 15.0	49.3 ± 23.8	4.7 ± 2.6	17.5 ± 3.5	6.0 ± 1.8	16.6 ± 4.6	0.6 ± 0.1
25 µM	1.9 ± 1.1	0.5 ± 0.3	0.4 ± 0.2	0.1 ± 0.04	1.1 ± 0.6	1.2 ± 0.1	0.5 ± 0.1	0.1 ± 0.01

Results for incubation for 48 h has a higher mitochondrial membrane potential than that 12 h due to the different life cycle of malaria and also increase in parasite growth in untreated condition. Since most of the parasite are in trophozoite or schizont and at 2% parasitaemia during seeding, so at 12 h, they will be in ring stage and parasitaemia may remained at 2%. While at 48 h, a full life cycle is back to the trophozoite or schizont stage and parasitaemia can be more than 2%

#### Type of cell death by morphological study

Definitive programme cell death in *P. falciparum* induced by anti-malarial drugs, unlike that in human cells, is still not conclusive [69]. *Plasmodium falciparum* in red blood cells treated with drugs like chloroquine are also known to result in autophagic-like death without chromatin condensation (apoptosis) or swelling and disruption of plasma membrane (necrosis) [70]. Although *P. falciparum* lacks classic caspases, it has been reported that it can produce meta-caspases that cause induction of programmed cell death [71]. Here, to conduct a preliminary investigation into the type of cell death of the *P. falciparum* strains treated with copper(II) complexes (**1**) and (**3**), Giemsa and Hoechst staining were used to examine morphological changes of the treated parasitic cells in the infected RBC.

#### Morphology examination (Giemsa)

**Fig. 8:**
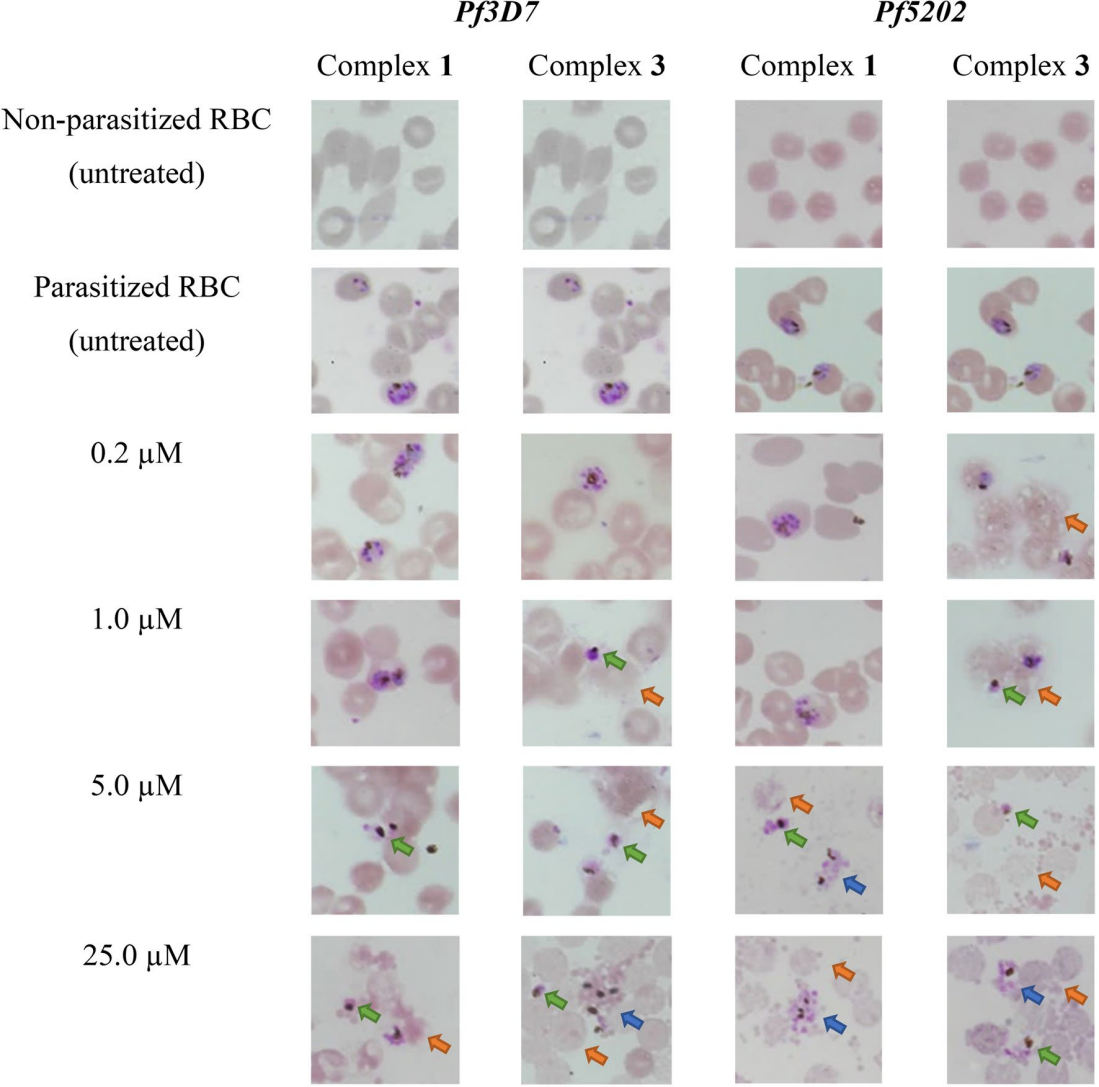
Morphology examination of *Pf3D7* and *Pf5202* on Giemsa-stained smear after treated with increasing concentration of (**1**) and (**3**) for 72 h at 100x (oil immersion) magnification. Blue arrow: ruptured of schizonts; green arrow: distorted and shrunk pyknotic form; orange arrow: haemolysis

Giemsa dye, a mixture of Azure, methylene blue and eosin, is commonly used nucleic acid stain that discriminates human RBC and malaria parasites [72]. It will stain human RBC, malarial cytoplasm and malarial nucleus (chromatin) pinkish-grey, purplish-blue and purplish-red respectively. *Pf3D7*- and *Pf5202*-infected RBC cells were treated with increasing concentrations (0.2, 1, 5 and 25 µM) of copper(II) complexes (**1**) and (**3**) for a period of 72 h. Images of untreated non-parasitized RBC (nRBC) and parasitized RBC (pRBC) as well as treated infected RBC were captured using bright field upright microscope at oil immersion 100 × magnification. Results are presented in Fig. 8.

Untreated nRBC were stained in pinkish-grey and no *P. falciparum* was observed. Untreated nRBC appeared to be healthy and rounded cells. Similar observation was noticed for untreated pRBC where all the untreated pRBC remained as healthy and rounded cells, and the morphology of *Pf3D7* and *Pf5202* was in good condition and had much greater parasitaemia. Majority (~ 90%) of the untreated *Pf3D7* and *Pf5202* was in the trophozoite and schizont forms, consisting of little double chromatin dot “headphone” form of rings. After treatment with (**1**) or (**3**) against pRBC, several morphological observations were spotted. The common abnormalities of RBC include haemolysis, membrane deformation and decrease in RBC density. As for the morphology of *Pf3D7* and *Pf5202*, decrease in parasitaemia, deformation of parasitic membrane, parasite shrinkage, rupture of schizont and formation of pyknotic (non-viable) form were noticed. All these abnormalities are the characteristics of apoptotic programmed cell death of RBC and *P. falciparum* [73]. Similar observations were reported by numerous researchers when *P. falciparum* was treated with chloroquine, atovaquone, synthetic florescence peptide, peptide-morpholino oligomer, endoperoxide compounds, Zn(II)-dipicolylamine complexes or dihydroartemisinin [74–79].

When *Pf3D7*-infected RBC was treated with (**1**) (IC_50_ = 3.51 µM), pRBC remained in healthy condition at 0.2 µM and 1 µM with the *P. falciparum* appearing mainly as trophozoites and schizonts. Between 1 µM and 25 µM, pRBC started to deform and rupture, leading to haemolysis. In turn, lysis of RBC indirectly reduced the parasitaemia of *Pf3D7* due to the lack of nutrients supplied. At this point, *Pf3D7* underwent pyknosis and the membrane of parasites were distorted, shrunk, and broken as shown in images of pRBC treated with 5 µM and 25 µM. Similar observations were seen when *Pf5202*-infected RBC was treated with (**1**) (IC_50_ = 17.87 µM). At 0.2 µM and 1 µM, *Pf5202* appeared as healthy schizonts. When pRBC cells were treated with concentrations greater than 1 µM, pRBC membrane deformation, haemolysis, decrease in parasitaemia, formation of pyknotic parasite, shrunken in size and rupture of schizonts were observed.

#### Morphology examination (Hoechst)

**Fig. 9:**
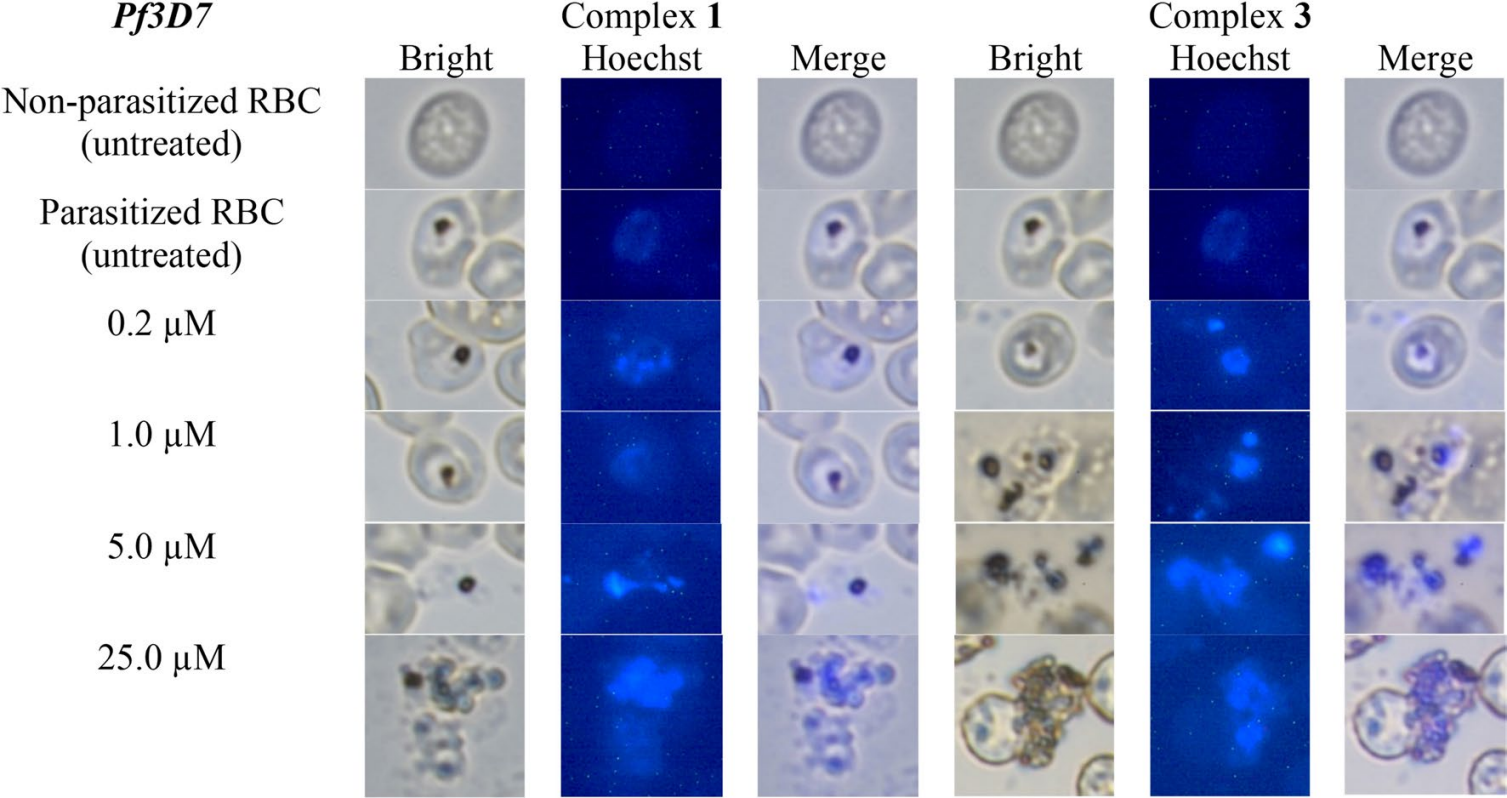
Nuclear morphological observation of *Pf3D7* using Hoechst stain after treated with increasing concentration of (**1**) and (**3**) for 72 h at 100x (oil immersion) magnification

**Fig. 10:**
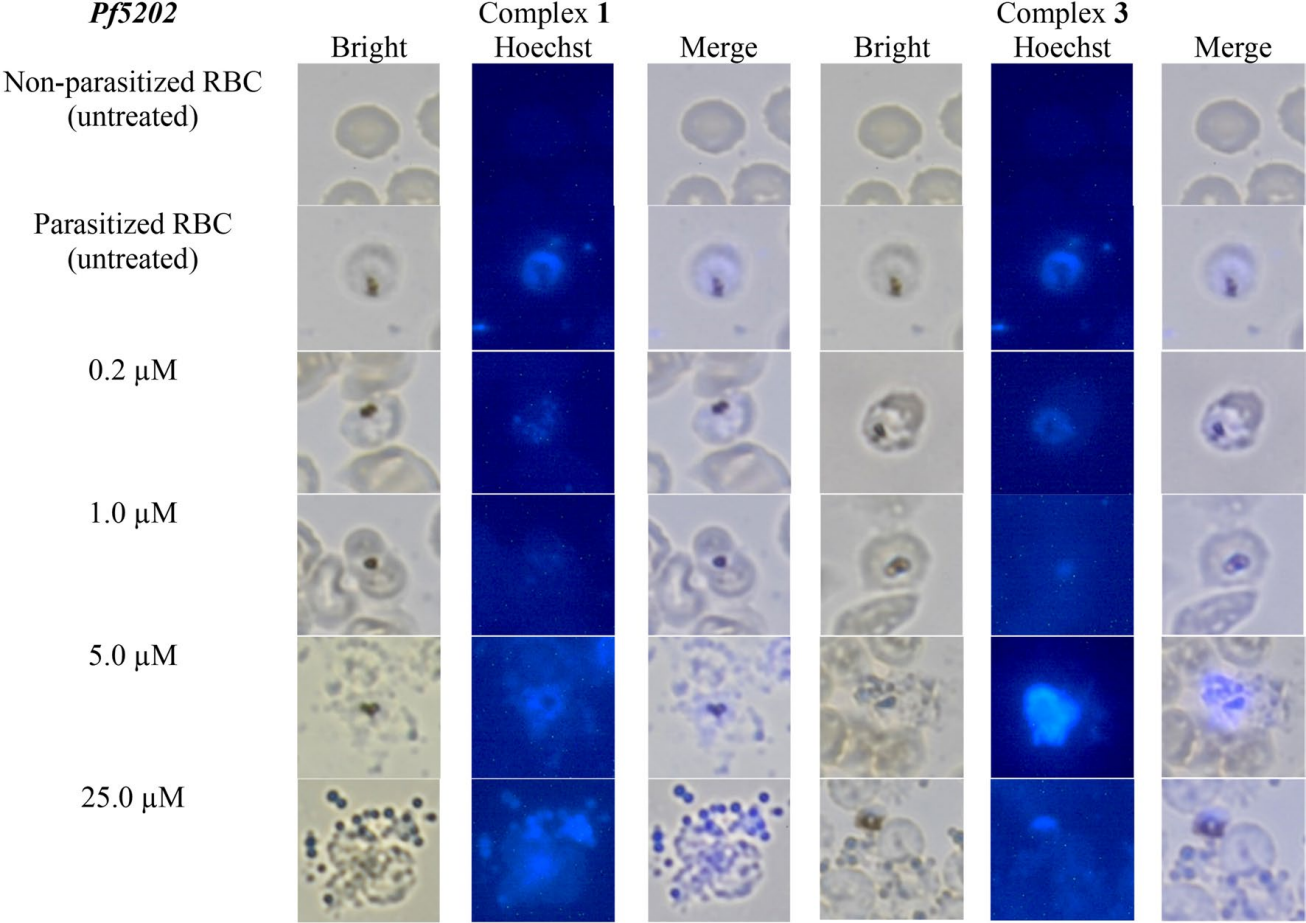
Nuclear morphological observation of *Pf5202* using Hoechst stain after treated with increasing concentration of (**1**) and (**3**) for 72 h at 100x (oil immersion) magnification

The morphological features of apoptosis of *P. falciparum* can be indicated by chromosome condensation and fragmentation of the *P. falciparum* nuclei in the non-nuclear RBC cells [73, 80]. The condensed chromosomes would be stained bright blue. The pRBC cells were treated with (**1**) and (**3**) respectively for 72 h, and the resultant pRBC were visualized and photographed at 100 × magnification with oil immersion. Typical images were enlarged to look at single cells and are depicted in Fig. 9 for *Pf3D7* and Fig. 10 for *Pf5202*. At 72 h incubation, *P. falciparum* were at mature trophozoite stage. The nuclei of *P. falciparum* trophozoites in pRBC were observed under brightfield microscope as dots, and they were stained brighter blue which increased in fluorescence intensity with increasing concentration of copper(II) complexes of (**1**) and (**3**) from 0.2, 1, 5 to 25 µM compared to the untreated pRBC. Since Hoechst stain is specific for chromatin state and DNA conformation, the increasingly stronger observed fluorescence intensity suggests more deformation and condensation of chromatin and DNA [81].

When *Pf3D7*-infected RBC cells were treated with (**1**) and (**3**), brighter fluorescence was observed for treatment with 1 µM or more of the dicopper complex (**3**) than that with treatment with (**1**). This suggests that this copper complex (**3**) (IC_50_, 0.90 µM) induced a stronger chromatin condensation than (**1**). The occurrence of chromatin condensation directly indicates the induction of apoptotic-like phenomenon. Thus, *Pf3D7* underwent apoptosis when treated with (**1**) and (**3**). Furthermore, distorted parasite, pyknotic forms and rupture of schizonts were seen at 5 µM and 25 µM for (**1**) and starting from 1 µM for (**3**). Similar phenomena were observed when *Pf5202* were treated with (**1**) and (**3**). Dicopper complex (**3**) (IC_50_, 2.21 µM) had stronger fluorescence intensity starting from 5 µM than mononuclear copper complex (**1**). This greater fluorescence could be explained by the fact that (**3**) is each a dinuclear copper(II) complex consisting of two copper(II) ions, and it was possible that double or faster generation of ROS could occur, resulting in more *P. falciparum* cells with stronger chromatin condensation. Besides that, distorted parasite, pyknotic forms and ruptured of schizonts were noticed at 5 µM and 25 µM. Overall, the results suggest that the tested copper(II) complexes could induced apoptosis of *P. falciparum*.

## Conclusion

Copper(II) complexes showed anti-malarial potency against both *Pf3D7* and *Pf5202* in sub-micromolar to micromolar range. The zinc(II) complexes were effective against *Pf3D7* with excellent therapeutic index but encountered total resistance against *Pf5202*. Among the four, the dinuclear copper(II) complex was the most potent against both strains. The zinc(II) complexes caused no haemolysis of RBC while copper(II) complexes induced increased haemolysis with increasing concentration. Further mechanistic studies of both copper(II) complexes on both *Pf3D7* and *Pf5202* strains showed induction of ROS, 20S malarial proteasome inhibition, loss of mitochondrial membrane potential and morphological features indicative of apoptosis. The dinuclear [Cu(phen)-4,4′-bipy-Cu(phen)](NO_3_)_4_ is highly potent and can overcome the total drug-resistance of *Pf5202* towards chloroquine and artemisinin. The other three copper(II) and zinc(II) complexes were only effective towards the drug-sensitive *Pf3D7*, with the latter causing no haemolysis of RBC.

## Supplementary Information

**Additional file 1: Table S1.** Crystallographic data for copper(II) complex (**3**). **Table S2.** Selected bond distances and bond angles for copper(II) complex (**3**).

## Data Availability

The datasets used and/or analysed during the current study are available from the corresponding author on request.

## Abbreviations

Δψm: Mitochondrial membrane potential
4,4′-bipy: 4,4′-bipyridine
ACTs: Artemisinin-combination therapies
ART: Artemisinin
CQ: Chloroquine diphosphate salt
Cu(NO_3_)•3H_2_O: Copper(II) nitrate trihydrate
IC_50_: Inhibitory concentration at 50% inhibition of growth
nRBC: Non-parasitized red blood cells
*Pf3D7*: *Plasmodium falciparum* 3D7
*Pf5202*: *Plasmodium falciparum* IPC5202
phen: 1,10-phenanthroline
pRBC: Parasitized red blood cells
RPMI: Roswell Park Memorial Institute
ROS: Reactive oxygen species
WHO: World Health Organization
Zn(NO_3_)•6H_2_O: Zinc(II) nitrate hexahydrate
