# Supplementary material for: Dinuclear and mononuclear metal(II) polypyridyl complexes against drug-sensitive and drug-resistant Plasmodium falciparum and their mode of action
Source: Malar J. 2022 Dec 17;21:386. doi: 10.1186/s12936-022-04406-0 (PMC9758846; doi:10.1186/s12936-022-04406-0)
Supplement: Supplementary file 1 — Additional file 1: Table S1. Crystallographic data for copper(II) complex (3). Table S2. Selected bond distances and bond angles for copper(II) complex (3). [file 12936_2022_4406_MOESM1_ESM.docx]

**Dinuclear and mononuclear metal(II) polypyridyl complexes against drug-sensitive and drug-resistant *Plasmodium falciparum* and their mode of action**

Jing Wei Lai^1^, Mohd Jamil Maah^1^, Kong Wai Tan^1^, Rozie Sarip^1^, Yvonne Ai Lian Lim^2^, Rakesh Ganguly^3^, Loke Tim Khaw^4^, Chew Hee Ng^4^*

**Supplementary Information**

**Table S1** Crystallographic data for (**3**).

| Compound | [Cu(phen)(NO_3_)-4,4’-bipy-Cu(phen)(NO_3_)](NO_3_)_2_ |
| --- | --- |
| Empirical formula | C_34_H_28_Cu_2_N_10_O_14_ |
| Formula weight | 927.76 |
| Crystal system | triclinic |
| Crystal size (mm) | 0.40 x 0.3 x 0.2 |
| Shape | block |
| Space group | P -1 |
| Unit cell dimensions (Å),(˚) | a = 7.3616 (4) α = 92.283 (5)  b = 8.9066 (5) β = 97.585 (5)  c = 13.7780 (10) γ = 96.287 (10) |
| Volume, Z (Å^3^) | 888.74 (10) |
| Ρ_calc_ (g cm^-3^) | 1.7333 |
| µ (cm^-1^) | 2.250 |
| *F* (000) | 472 |
| Theta range of data collection | 11.0650 – 70.9520 |
| Limiting frequency | -8 ≤ h ≤ 8  -10 ≤ k ≤ 10  -16 ≤ l ≤ 16 |
| Total reflections | 3379 |
| Independent reflections | 3379 |
| Refinement method | Full matrix least squares n F^2^ |
| Data/restraints/parameters | 379/2/278 |
| Goodness of fit | 1.085 |
| Final R indices [*I* > 2(*l*)] | R1 = 0.0397, wR2 = 0.1150 |
| R indices (all data) | R1 = 0.0477, wR2 = 0.1589 |
| Largest diff. peak & hole (e Å^3^) | 0.38 and -0.51 |

**Table S2** Selected bond distances and bond angles for (**3**).

| **Bond distance (Å)** | | | |
| --- | --- | --- | --- |
| Cu1 – N1 (amino, phen) | 2.028(3) | Cu1 – O1 (OH_2_) | 2.224(2) |
| Cu1 – N2 (amino, phen) | 2.016(3) | Cu1 – O6 (ONO_2_) | 1.994(2) |
| Cu1 – N3 (amino, 4,4’-bipy) | 1.986(3) | Cu1 – O5 (ONO_2_) | 2.707(3) |
| **Bond angle (˚)** | | | |
| N1 – Cu1 – O6 | 161.91(9) | N2 – Cu1 – O1 | 86.95(10) |
| O1 – Cu1 – O6 | 91.95(10) | N3 – Cu1 – O6 | 93.46(11) |
| O1 – Cu1 – N1 | 104.28(10) | N3 – Cu1 – N1 | 92.01(10) |
| N2 – Cu1 – O6 | 91.55(10) | N3 – Cu1 – O1 | 98.51(11) |
| N2 – Cu1 – N1 | 81.54(10) | N3 – Cu1 – N2 | 172.46(11) |

**Table S3** Molar conductivity of 1 mM of aqueous solutions of (**1**) – (**4**) and its precursors at room temperature.

| **Compound**  **(Solution used)** | **Molar conductivity ʌ_m_ (S cm^2^ mol^-1^)** | | | | **Postulated ion in aqueous solution** |
| --- | --- | --- | --- | --- | --- |
|  | **0 h** | **24 h** | **48 h** | **72 h** |  |
| phen (H_2_O-EtOH) | 2.7 ± 0.3 | 3.6 ± 0.2 | 4.7 ± 0.2 | 5.3 ± 0.3 | phen |
| 4,4’-bipy (H_2_O-EtOH) | 7.1 ± 0.3 | 7.8 ± 0.2 | 8.5 ± 0.2 | 9.3 ± 0.2 | 4,4’-bipy |
| Cu(NO_3_)_2_•3H_2_O (H_2_O) | 252.5 ± 7.3 | 253.5 ± 7.3 | 254.4 ± 8.0 | 252.9 ± 8.6 | [Cu(H_2_O)_6_]^2+^, 2NO_3_^-^ |
| Zn(NO_3_)_2_•6H_2_O (H_2_O) | 239.4 ± 5.1 | 238.1 ± 4.4 | 238.8 ± 4.2 | 238.3 ± 5.2 | [Zn(H_2_O)_6_]^2+^, 2NO_3_^-^ |
| **1** (H_2_O) | 270.9 ± 12.2 | 270.6 ± 14.9 | 271.8 ± 14.8 | 271.2 ± 14.2 | [Cu(phen)_2_(H_2_O)]^2+^, 2NO_3_^-^ |
| **2** (H_2_O) | 215.4 ± 3.1 | 215.7 ± 3.4 | 216.5 ± 3.0 | 217.0 ± 2.5 | [Zn(phen)_2_(H_2_O)_2_]^+^_,_ 2NO_3_^-^ |
| **3** (H_2_O-DMSO)* | 350.3 ± 0.7 | 351.9 ± 0.2 | 350.7 ± 0.4 | 352.5 ± 0.6 | [Cu(phen)(H_2_O)(NO_3_)-4,4’-bipy-Cu(phen)(H_2_O)(NO_3_)]^2+^, 2NO_3_^-^ |
| **4** (H_2_O-DMSO)* | 363.7 ± 0.5 | 365.6 ± 0.2 | 365.2 ± 0.4 | 366.4 ± 0.4 | [Zn(phen)(H_2_O)(NO_3_)-4,4’-bipy-Zn(phen)(H_2_O)(NO_3_)]^2+^, 2NO_3_^-^ |
| *For 1 mM complexes (**3**) and (**4**): (10% DMSO). | | | | | |

**Table S4** UV-visible data (molar absorptivity) of (**1**) – (**4**) and its precursors at room temperature.

| **Compound** | **Molar absorptivity, λ_max_/nm (ε/mol^-1^dm^3^cm^-1^)** | | | |
| --- | --- | --- | --- | --- |
|  | **0 h** | **24 h** | **48 h** | **72 h** |
| phen | 232 (30667)  266 (25000) | 232 (40000)  264 (40000) | 232 (38667)  264 (41333) | 232 (32333)  264 (34000) |
| 4,4’-bipy | 240 (15333)  272 (6667) | 239 (8333)  272 (3000) | 240 (15000)  272 (6667) | 240 (14333)  272 (6333) |
| Cu(NO_3_)_2_·3H_2_O | 204 (16667)  810 (10) | 207 (8333)  810 (10) | 203 (21667)  810 (10) | 203 (21000)  810 (10) |
| Zn(NO_3_)_2_·6H_2_O | 205 (25667) | 205 (25667) | 201 (31000) | 201 (29333) |
| **1** | 205 (58333)  222 (51000)  271 (46667)  294 (18000)  709 (64) | 207 (50667)  222 (44333)  271 (41667)  294 (16000)  709 (66) | 203 (63667)  222 (55667)  271 (49667)  294 (18000)  709 (64) | 207 (59333)  222 (51667)  271 (46333)  294 (17000)  709 (66) |
| **2** | 201 (55333)  223 (49333)  270 (45000)  292 (17667) | 201 (44667)  223 (41333)  270 (39000)  292 (15667) | 201 (60667)  223 (53667)  270 (48000)  292 (18333) | 201 (57333)  223 (50667)  270 (45667)  292 (17667) |
| **3** | 230 (65000)  272 (66667)  294 (19333)  705 (72) | 230 (63000)  272 (65000)  294 (18667)  705 (72) | 230 (59333)  272 (62333)  294 (17667)  705 (70) | 230 (60333)  272 (62333)  294 (17667)  705 (70) |
| **4** | 231 (78333)  270 (71333)  292 (22667) | 231 (76667)  270 (70333)  292 (21667) | 231 (73333)  270 (67333)  292 (20667) | 231 (72667)  270 (67667)  292 (20667) |

**Table S5** ESI-MS data of complexes (**1**) – (**4**)

| **Compound** | **Found m/z**  **(Calculated m/z)** | **Intensity (%)** | **Species** |
| --- | --- | --- | --- |
| **1** | 484.8  (485.3)  486.7  (487.1) | 100  49 | [^63^Cu(phen)_2_(NO_3_)]^+^  [^65^Cu(phen)_2_(NO_3_)]^+^ |
| **2** | 485.9  (486.3)  487.9  (488.3) | 67  40 | [^64^Zn(phen)_2_(NO_3_)]^+^  [^66^Zn(phen)_2_(NO_3_)]^+^ |
| **3** | 237.9  (237.8)  238.8  (239.2) | 100  56 | [^63^Cu(phen)-4,4’-bipy-^63^Cu(phen)(NO_3_)+3H]^3+^  [^65^Cu(phen)-4,4’-bipy-^63^Cu(phen)(NO_3_)+3H]^3+^ |
|  | 382.2  (382.2)  384.1  (384.2) | 31  16 | [^63^Cu(phen)(NO_3_)-4,4’bipy-^63^Cu(phen)(NO_3_)-H]^2+^  [^65^Cu(phen)(NO_3_)-4,4’bipy-^63^Cu(phen)(NO_3_)-H]^2+^ |
| **4** | 383.6  (384.2)  385.6  (386.2) | 100  62 | [^64^Zn(phen)(NO_3_)-4,4’-bipy-^64^Zn(phen)(NO_3_)]^2+^  [^66^Zn(phen)(NO_3_)-4,4’-bipy-^64^Zn(phen)(NO_3_)]^2+^ |
|  | 234.5  (235.5)  238.4  (236.8) | 55  50 | [^64^Zn(phen)-4,4’-bipy-^64^Zn(phen)(NO_3_)]^3+^  [^66^Zn(phen)-4,4’-bipy-^64^Zn(phen)(NO_3_)]^3+^ |


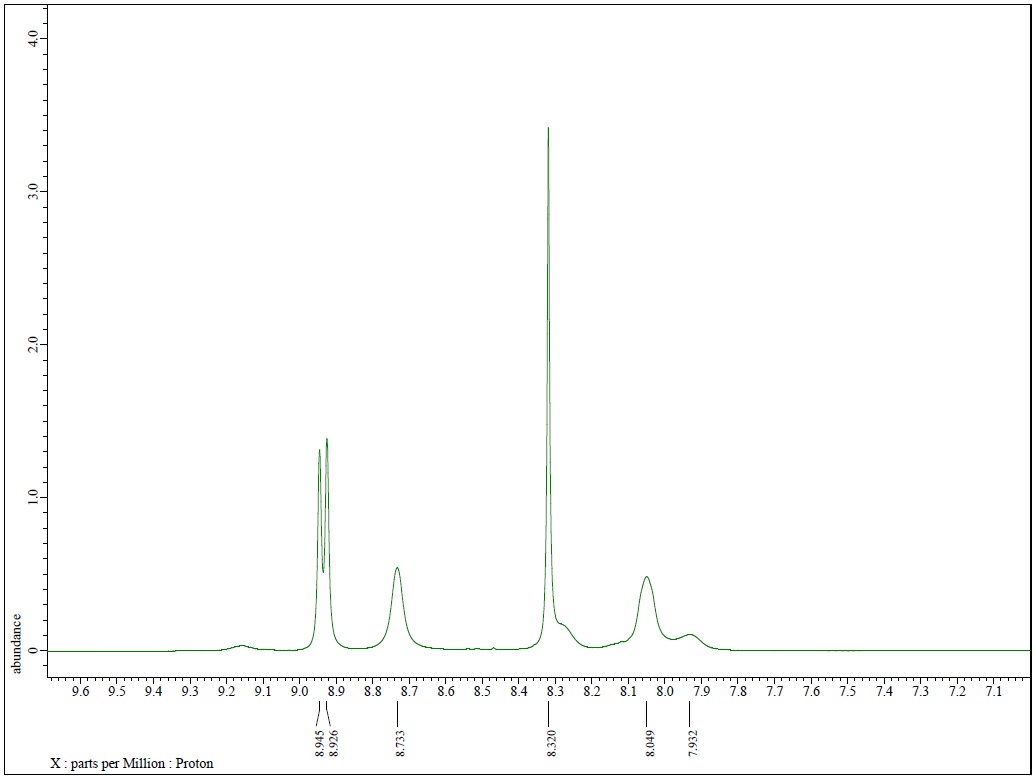


*b*

*d*

*c*

*a*

**Fig. S1.1** ^1^H NMR spectrum of (**2**). ^1^H NMR (400 MHz, DMSO-d, δ ppm): 8.049 (4H, t, Ar-H); 8.320 (4H, s, Ar-H); 8.733 (4H, d, Ar-H); 8.945 (4H, d, Ar-H).


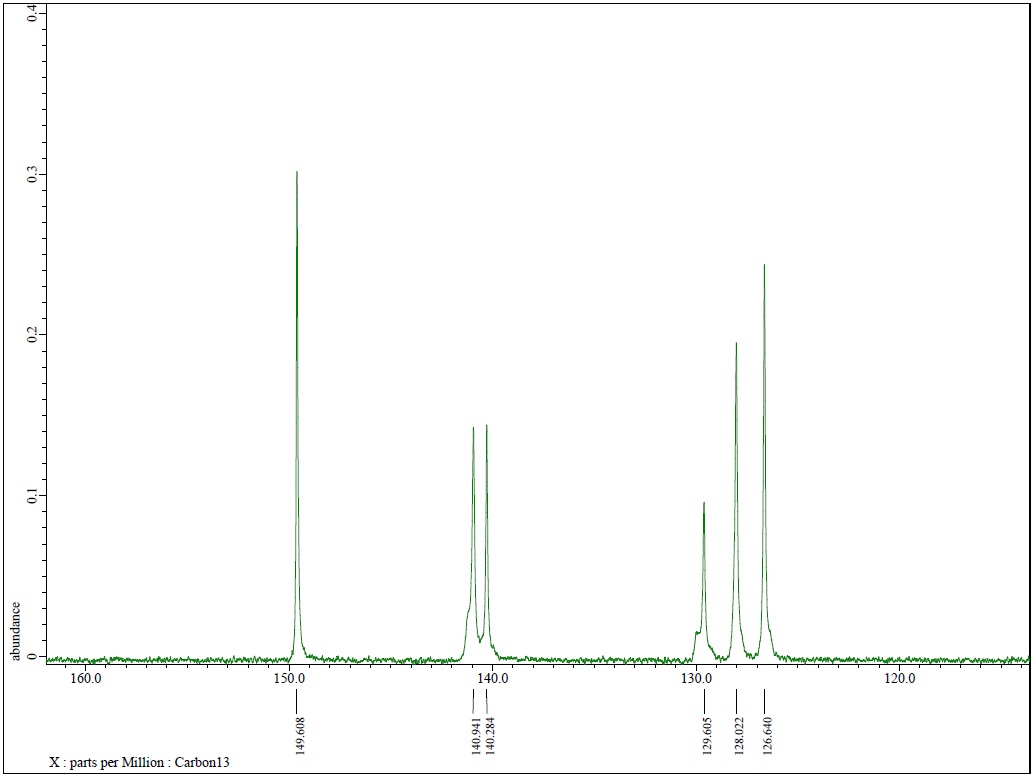


*f*

*d*

*e*

*c*

*b*

*a*

**Fig. S1.2** ^13^C NMR spectrum of (**2**). ^13^C NMR (400 MHz, DMSO-d, δ ppm): 126.640 (C_Ar_); 128.022 (C_Ar_); 129.605 (C_Ar_); 140.284 (C_Ar_); 140.941 (-C_Ar_=N); 149.608 (-C_Ar_-N).

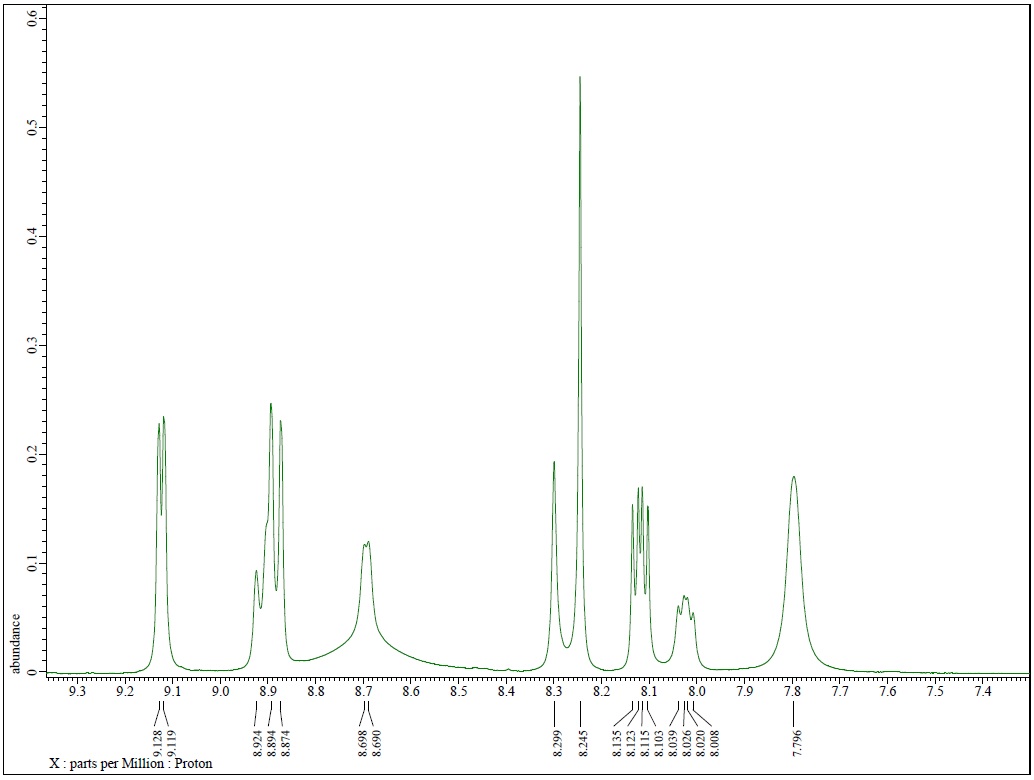


*a*

*d*

*f*

*e*

*c*

*b*

**Fig. S2.1** ^1^H NMR spectrum of (**4**). ^1^H NMR (400 MHz, DMSO-d, δ ppm): 7.396 (4H, d, Ar-H); 8.123 (4H, t, Ar-H); 8.245 (4H, s, Ar-H); 8.698 (4H, d, Ar-H); 8.894 (4H, t, Ar-H); 9.128 (4H, d, Ar-H).

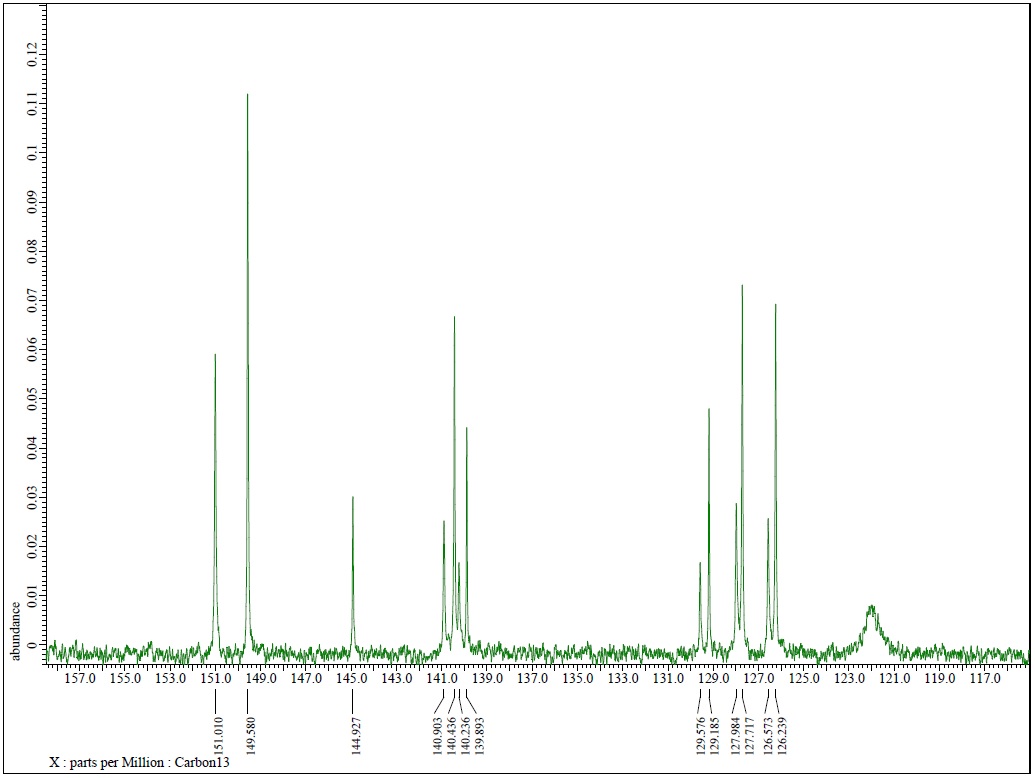


*a*

*h*

*c*

*d*

*f*

*g*

*i*

*b*

*e*

*d*

**Fig. S2.2** ^13^C NMR spectrum of (**4**). ^13^C NMR (400 MHz, DMSO-d, δ ppm): 126.219-129.576 (C_Ar_); 139.893-140.436 (C_Ar_); 140.903 (=C_Ar_-N); 144.927 (-C_Ar_-N); 149.580 (-C_Ar_-C_Ar_); 151.010 (-C_Ar_=N).
